# FOXL2 and FOXA1 cooperatively assemble on the *TP53* promoter in alternative dimer configurations

**DOI:** 10.1093/nar/gkac673

**Published:** 2022-08-03

**Authors:** Yuri Choi, Yongyang Luo, Seunghwa Lee, Hanyong Jin, Hye-Jin Yoon, Yoonsoo Hahn, Jeehyeon Bae, Hyung Ho Lee

**Affiliations:** Department of Chemistry, College of Natural Sciences, Seoul National University, Seoul 08826, Korea; School of Pharmacy, Chung-Ang University, Seoul 06974, Korea; Department of Life Science, Chung-Ang University, Seoul 06974, Korea; Key Laboratory of Natural Medicines of the Changbai Mountain, Ministry of Education, College of Pharmacy, Yanbian University, Yanji 133002, Jilin Province, China; Department of Chemistry, College of Natural Sciences, Seoul National University, Seoul 08826, Korea; Department of Life Science, Chung-Ang University, Seoul 06974, Korea; School of Pharmacy, Chung-Ang University, Seoul 06974, Korea; Department of Chemistry, College of Natural Sciences, Seoul National University, Seoul 08826, Korea

## Abstract

Although both the p53 and forkhead box (FOX) family proteins are key transcription factors associated with cancer progression, their direct relationship is unknown. Here, we found that FOX family proteins bind to the non-canonical homotypic cluster of the p53 promoter region (*TP53*). Analysis of crystal structures of FOX proteins (FOXL2 and FOXA1) bound to the p53 homotypic cluster indicated that they interact with a 2:1 stoichiometry accommodated by FOX-induced DNA allostery. In particular, FOX proteins exhibited distinct dimerization patterns in recognition of the same p53-DNA; dimer formation of FOXA1 involved protein–protein interaction, but FOXL2 did not. Biochemical and biological functional analyses confirmed the cooperative binding of FOX proteins to the *TP53* promoter for the transcriptional activation of *TP53*. In addition, up-regulation of *TP53* was necessary for FOX proteins to exhibit anti-proliferative activity in cancer cells. These analyses reveal the presence of a discrete characteristic within FOX family proteins in which FOX proteins regulate the transcription activity of the p53 tumor suppressor via cooperative binding to the *TP53* promoter in alternative dimer configurations.

## Graphical Abstract

**Graphical Abstract:**
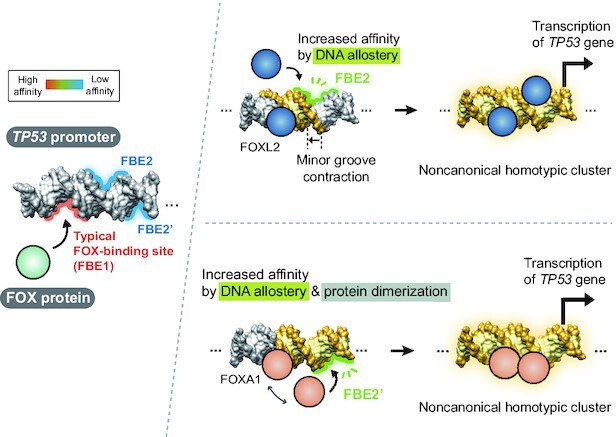
Cooperative regulation of non-canonical homotypic cluster by Forkhead-box family.

## INTRODUCTION

The p53 protein is one of the most studied tumor suppressor proteins in cancer research as a major cellular ‘gatekeeper’ that inhibits development of a variety of tumors (1) by transcriptional regulation of downstream target genes involved in apoptosis, cell cycle arrest, senescence, DNA repair and metastasis (2). The tumor suppressor p53 is lost or mutated in approximately half of all human cancers, and > 80% of the mutated nucleotides are located in the p53 DNA-binding domain, which abrogates p53 transcriptional activity (3). p53 itself is also transcriptionally controlled, and several transcription factor-binding motifs are conserved in the *TP53* gene promoter region. Various transcription factors, such as AP-1, NFκB, Pax proteins, IFNα/β and the CAAT/enhancer-binding protein (C/EPB) family have been reported to activate the promoter of the human p53 gene (*TP53*) (4–6). However, many studies have focused on identifying p53 target genes, and thus the upstream molecular mechanisms that control transcription of the *TP53* gene remain largely unknown.

Forkhead box (FOX) proteins belong to an evolutionarily conserved family of transcription factors expressed from yeast to humans, and possess a highly conserved DNA-binding domain (DBD), called the ‘forkhead box’ domain. Fifty FOX genes have been identified in the human genome, and these are classified into 19 FOX gene subfamilies (FOXA to FOXS) based on their sequence homology (7–10). Most FOX proteins bind to the typical FOX DNA-binding element (FBE) 5′-RYAAAYA-3′ (R = A or G, Y = C or T) to regulate the transcription of their target genes (11). They play pivotal roles in normal development processes and maintenance of cellular homeostasis, including cell migration, differentiation, proliferation and apoptosis (12–14). More recently, FOX proteins have emerged as critical transcriptional regulators in cancer-related processes such as tumorigenesis and cancer progression (9,15,16). Interestingly, previous structural studies reported highly cooperative dimerization characteristics of FOX proteins on DIV motifs (‘DIV’ for diverging half-sites) (17–20).

Members of the FOX family of proteins tend to exhibit tissue-specific expression patterns and functions. For instance, forkhead box L2 (FOXL2) plays a pivotal role in the development and maintenance of the ovary (21,22). FOXL2 regulates the transcription of anti-Müllerian hormone, which is essential for proper ovarian follicle development (23,24). Germline FOXL2 mutation is related to blepharophimosis–ptosis–epicanthus inversus syndrome (BPES), which results in eyelid defects and premature ovarian failure (POF) (25). In particular, a somatic mutation in the FOXL2 gene (c.402C>G; Cys134Trp) is known to be the only mutation related to adult-type granulosa cell tumors, and was present in 97% of patients (26–28).

In this study, we revealed the unexplored transcriptional regulation of p53 by the large family of FOX transcription factors. Unexpectedly, we identified a non-canonical homotypic cluster region of *TP53*, where FOX proteins bind to play a role as transcription regulators of p53. Crystal structures of FOX proteins (FOXL2 and FOXA1) bound to the *TP53* promoter showed that FOX proteins bind to the homotypic cluster in the *TP53* promoter with a 2:1 stoichiometry, revealing the presence of additional FOX-binding elements adjacent to the typical FBE. When one FOX protein binds to the typical FBE 5′-RYAAAYA-3′ (R = A or G, Y = C or T) of the *TP53* promoter, the minor groove width becomes narrower to create a new binding site for another FOX protein by increasing its binding affinity via DNA allostery. Accordingly, we confirmed *TP53* as a novel target of FOX proteins using cell biology studies. These studies provide structural insights into DNA recognition of FOX proteins and, more importantly, clues to understand the molecular mechanism through which FOX proteins act cooperatively to regulate *TP53* transcription.

## MATERIALS AND METHODS

### Plasmids

pCMV-Myc-FOXL2 was generated as previously described (29). The pGL2-p53-Luc plasmid (30) was a generous gift from Dr Hur Man-Wook (Yonsei University, Seoul, Korea). Utilizing pGL2-p53-Luc as a template, the full-length (from –1800 to –1 bp) and truncated (from –551 to –1 bp) human *TP53* promoters were amplified by polymerase chain reaction (PCR) using the following primers: -1800F with -1800R and -551F with -551R, respectively. The amplified PCR products were digested with KpnI and NheI (Takara Bio, Shiga, Japan) and ligated into the pGL4.10 empty vector (Promega, Madison, WI, USA). pGL4.10-*TP53* mutant constructs were generated by recombinant PCR using the following primers: TP53-mut1-F, TP53-mut1-R, TP53-mut2-F, TP53-mut2-R, TP53-mut3-F and TP53-mut3-R. pCMV-Myc-FOXL2 mutants were generated by recombinant PCR using the following primers: S58A-F and S58A-R; Y59A-F and Y59A-R; Y81A-F and Y81A-R; N100A-F and N100A-R; S101A-F and S101A-R; R103A-F and R103A-R; H104A-F and H104A-R; N105A-F and N105A-R; S107A-F and S107A-R; L108A-F and L108A-R; K124A-F and K124A-R; Helix 1-F and Helix 1-R; and helix 3-F and helix 3-R. p3XFLAG-CMV-10-FOXL2, FOXA1 and FOXO3 were produced by PCR using pCMV-Myc-FOXL2, pBluescriptR-FOXA1 (Clone ID: hMU002984, Korean Human Gene Bank, Daejeon, Korea) and pOTB7-FOXO3 (Clone ID: hMU010340, Korean Human Gene Bank) as a template, respectively. The following primers were used: FOXL2-F, FOXL2-R, FOXA1-F, FOXA1-R, FOXO3-F and FOXO3-R. FOXL2 and FOXA1 PCR products were digested with EcoRI and KpnI (Takara Bio), FOXO3 was digested with KpnI and BamHI (Takara Bio) and ligated into p3XFLAG-CMV-10 (Sigma-Aldrich). p3XFLAG-CMV-10-FOXA1 mutants were generated by recombinant PCR using the following primers: Y173A, S174A, S177A-F with Y173A, S174A, S177A-R; ΔFH-N-F with ΔFH-N-R; and ΔFH-C-F with ΔFH-C-R. The sequences of all primers are listed in Supplementary Table S1.

### Cell culture and transfection

HeLa human cervical carcinoma cells (Korean Cell Line Bank, Seoul, Korea), SiHa human cervical carcinoma cells (Korean Cell Line Bank) and 293T human embryonic kidney cells (ATCC, Manassas, VA, USA) were cultured in Dulbecco’s modified Eagle’s medium (DMEM) supplemented with 10% fetal bovine serum (FBS; Caisson, North Logan, UT, USA) and 1% penicillin–streptomycin (Caisson) at 37°C with 5% CO_2_. Human adult-type GCT-derived KGN cells (Riken, Tsukuba, Japan) were cultured in DMEM/F12 supplemented with 10% FBS and 1% penicillin–streptomycin. FOXL2 knockout (KO) cells were generated as previously reported (29) and cultured in the same manner as KGN cells. 293T cells were transfected using polyethylenimine (PEI) (Polysciences Inc., Warrington, PA, USA). KGN, HeLa and SiHa cells were transfected with Lipofectamine 3000 (Invitrogen, Carlsbad, CA, USA) according to the manufacturer's instructions.

### Luciferase assay

Luciferase activity was assessed as described previously (31). In brief, HeLa cells were co-transfected with 500 ng of TP53-luciferase reporter, 100 ng of pCMV β-galactosidase (Clontech, Mountain View, CA, USA) and the indicated amounts of plasmids as well as small interfering nucleotides. After 24 h of incubation, reporter activity was assessed using the Luciferase Assay System Kit (Promega, Madison, WI, USA). Absorbance and luminescence were measured using a FlexStation3 Microplate Reader (Molecular Devices, Eugene, OR, USA).

### Small RNA interference

Small interfering RNA (siRNA) target sequences against FOXL2 (5′-GGCAUCUACCAGUACAUCA-3′), siTP53 (5′-CACUACAACUACAUGUGUA-3′) and negative control siRNA (SN-1003) were purchased from Bioneer (Daejeon, South Korea). Cells were transfected with siRNAs using Lipofectamine 3000 (Invitrogen), according to the manufacturer's instructions.

### RNA extraction and real-time PCR

Total RNA was extracted using the TRIzol reagent (Invitrogen). The extracted RNA was analyzed by real-time PCR, as previously reported (31). In brief, the concentration and quality of RNA were determined using an ND-1000 spectrophotometer (NanoDrop, Waltham, MA, USA). Reverse transcription to cDNA was performed using the iScript™ Select cDNA Synthesis kit (Bio-Rad Laboratories, Hercules, CA, USA). All cDNAs used in real-time PCR was normalized to the expression level of glyceraldehyde phosphate dehydrogenase (GAPDH). Quantitative real-time PCR was performed using an iQ™ SYBR Green Supermix (Bio-Rad Laboratories). Real-time PCR was performed in a CFX-96TM thermal cycler and detection system (Bio-Rad Laboratories), and gene expression was quantified by the delta-delta-CT method. The nucleotide sequences of the primers used for real-time PCR (Bioneer) are listed in Supplementary Table S1.

### Generation of the cell line stably overexpressing FOXL2

All third-generation lentiviral plasmids were obtained from Addgene (Watertown, MA, USA). The envelope (pMD2.G; Addgene plasmid #12259; http://n2t.net/addgene:12259;RRID: Addgene_12259) and packaging plasmids (pRSV-Rev; Addgene plasmid #12253; http://n2t.net/addgene:12253;RRID: Addgene_12253 and pMDLg/pRRE; Addgene plasmid # 12251, http://n2t.net/addgene:12251;RRID: Addgene_12251) were gifts from Dr Tronoa (32). The transfer plasmid was a gift from Ie-Ming Shih (33) (pLenti-puro; Addgene plasmid #39481; http://n2t.net/addgene:39481;RRID: Addgene_39481). The pLenti-puro FLAG-tagged FOXL2 was generated by PCR using pcDNA3 FLAG-tagged FOXL2 as the template and the primers FLAG-FOXL2-F and FLAG-FOXL2-R. The PCR products were digested with BamHI and XbaI (Takara Bio) and ligated into the pLenti-puro vector (Addgene). 293T cells were used for lentivirus production by transfection of 12 μg of PMDLg/pRRE and pRSV-Rev, 8 μg of pMD2.G and 16 μg of pLenti-puro empty vector or pLenti-puro-FLAG-FOXL2 for 48 h. Viral supernatant was collected for KGN cell infection in the presence of 10 μg/ml polybrene (H9268, Sigma-Aldrich) (0.5 ml of lentivirus to 5 × 10^4^ cells in 1.5 ml of medium). After 48 h of infection, cells were selected with 2 μg/ml puromycin (P8833, Sigma-Aldrich) for 30 days.

### Immunoblot analysis

After treatment, the cells were harvested, lysed and their proteins subjected to sodium dodecyl sulfate–polyacrylamide gel electrophoresis (SDS–PAGE) for immunoblotting with the respective antibodies. The protein bands were detected using an Amersham Imager 600 (GE Healthcare Life Sciences, Amersham, Buckinghamshire, UK). The following antibodies were used in this study: anti-p53 (sc-126, Santa Cruz, CA, USA), anti-FOXL2 [generated in our laboratory as previously described (29)], anti-GAPDH (sc-47724, Santa Cruz), anti-Myc (#2276S, Cell Signaling Technology, Danvers, MA, USA), anti-FLAG (F1804, Sigma-Aldrich), anti-p21 (sc-397, Santa Cruz), anti-PARP1 (sc-74469, Santa Cruz), anti-BAX (sc-493, Santa Cruz) and anti-Caspase 3 (9662, Cell Signaling Technology).

### Chromatin immunoprecipitation (ChIP) analysis

ChIP assays were performed as described previously (33). Briefly, cells were transfected with empty vector or Myc-FOXL2 expression plasmid for 24 h. Cell lysates were immunoprecipitated using anti-Myc followed by purification of FOXL2-bound DNA. The differences in FOXL2-bound DNA were quantified by real-time PCR using the following primer set flanking the FOXL2-binding element in the *TP53* promoter: FBE-F (5′-TCTCATTCTCCAGGCTTCAGA-3′) and FBE-R (5′-TAGAATTTTTCTACTATCTTA-3′).

### Protein expression and purification

A construct comprising residues 52–148 of human FOXL2 (FOXL2-DBD) was cloned into the pGST2 vector (34), which introduced an N-terminal glutathione *S*-transferase (GST) tag. Primer sequences used for cloning are listed in Supplementary Table S1. The FOXL2-DBD was expressed in *Escherichia coli* BL21 (DE3) cells induced with 0.5 mM isopropyl-β-d-1-thiogalactopyranoside (IPTG) at 18°C for 18 h, followed by growth to mid-log phase at 37°C. For cell lysis, cell pellets were resuspended in buffer A (20 mM Tris–HCl pH 8.0 and 200 mM NaCl) containing 1 mM phenylmethylsulfonyl fluoride. Cells were lysed with a microfluidizer (Microfluidics, Westwood, MA, USA), and the lysed cells were centrifuged at 4600 × *g* (Vision V506CA rotor) for 30 min at 277 K to pellet the cell debris. Proteins were purified using a glutathione–Sepharose column (GE Healthcare), cleaved by TEV protease and further purified using cation-exchange chromatography (HiTrap SP HP, Cytiva, Marlborough, MA, USA). The eluate was purified by gel filtration on a HiLoad 16/60 Superdex 75 column (GE Healthcare) that had been pre-equilibrated with buffer containing 20 mM HEPES pH 7.5, 150 mM NaCl and 10 mM MgCl_2_. Peak fractions containing the FOXL2-DBD protein were pooled and concentrated to 10 mg/ml for crystallization. Constructs encoding FOXA1-DBD (residues 168–264) and FOXO3-DBD (residues 155–251) were also cloned into the pGST2 vector. The proteins were expressed and purified as described for FOXL2-DBD.

### DNA oligo preparation

All DNA oligonucleotides were purchased from Macrogen (Seoul, Korea). For crystallization, Daf-16 family binding element 2 (DBE2) DNA and p53-DNA oligonucleotides were resuspended to 100 μM in annealing buffer containing 10 mM Tris–HCl pH 8.0 and 50 mM NaCl, and annealed by mixing the complementary sequences in a 1:1 ratio. The mixtures were heated to 94°C for 5 min and slowly cooled to 296 K for 4 h. Duplex DNA used for isothermal titration calorimetry (ITC) was purchased from Macrogen (Seoul, Korea). For electrophoretic mobility shift assay (EMSA), DNA oligonucleotides labeled with 5′ Cy3 were resuspended at 100 μM in the annealing buffer and annealed by mixing the complementary sequences at a 1:1.1 ratio. The annealing steps were the same as those described above. The nucleotide sequences used in this study are listed in Supplementary Table S2.

### Crystallization and data collection

FOXL2–DNA complexes were prepared by mixing FOXL2-DBD protein with each DNA at a molar ratio of 1:1.2, and the mixture was incubated on ice for 30 min. The final concentration for crystallization was 5 mg/ml for both the DBE2 DNA complex and the p53-DNA complex. For FOXA1-DBD:p53-DNA, purified FOXA1-DBDs were mixed with p53 DNA at a molar ratio of 1:2, and incubated on ice for 30 min. The final concentration for crystallization was 10 mg/ml for both complexes. Crystals of FOXL2-DBD and the DBE2 DNA complex were grown at 291 K by the hanging-drop vapor diffusion method by mixing equal volumes of the protein solution (0.8 μl) and reservoir solution (0.8 μl), and the FOXL2-DBD crystals in complex with DBE2 DNA were formed in 0.1 M Bis-Tris pH 5.5, 0.15 M NaCl and 26% polyethylene glycol (PEG) 3350. Crystals were cryoprotected in a reservoir solution supplemented with 10% (v/v) glycerol. The crystals of the FOXL2-DBD:p53-DNA complex were formed in a reservoir solution containing 6% ethylene glycol, 0.1 M citric acid pH 3.5 and 15% PEG 6000; crystals were cryoprotected in a reservoir solution supplemented with 5% (v/v) glycerol. Crystals of the FOXA1-DBD:p53-DNA complex were grown at 291 K by the sitting-drop vapor diffusion method by mixing equal volumes of the protein solution (0.4 μl) and reservoir solution (0.4 μl). Crystals were formed in 0.1 M MES pH 6.0, 6% Tacsimate pH 6.0 and 20% PEG 4000, and cryoprotected in the reservoir solution supplemented with 20% (v/v) glycerol. All crystals were flash-frozen in a nitrogen gas stream at 100 K. Native data for the FOXL2-DBD:DBE2 DNA, FOXL2-DBD:p53-DNA and FOXA1-DBD:p53-DNA complexes were collected at 3.1, 3.15 and 2.1 Å resolution, respectively, using the EIIGER9M detector at the beamline 5C of Pohang Light Source (PLS). X-ray diffraction data for all crystals were processed and scaled using the program suite HKL2000 (35). Data collection statistics are summarized in Supplementary Table S3.

### Structure determination and refinement

Crystal structures of FOXL2-DBD in complex with the DBE2 DNA and p53-DNA were determined by molecular replacement using the software MOLREP (36), with FOXG1–DNA complex (PDB code 7CBY) (37) and FOXK1a–DNA complex (PDB code 2C6Y) (38), respectively, as a search model. For FOXA1-DBD:p53-DNA complex, the FOXG1–DNA complex (PDB code 7CBY) (37) was used as the search model. Manual model building was performed using the software COOT (39) and models were refined using the software PHENIX (40). Several rounds of model building, XYZ positional refinement and individual B-factor refinement were performed. The stereochemistry of the refined models was assessed using MolProbity (41). For the FOXL2-DBD:p53-DNA complex, the diffraction data were detwinned during the refinement process. Crystallographic and refinement statistics are summarized in Supplementary Table S3. The atomic coordinates and structural factors of three crystal structures of FOXL2-DBD in complex with the DBE2 DNA and the p53-DNA and of FOXA1-DBD in complex with p53-DNA have been deposited in PDB (PDB ID codes 7VOU, 7VOV and 7VOX, respectively).

### Isothermal titration calorimetry

ITC experiments were performed using Affinity ITC instruments (TA Instruments, New Castle, DE, USA) at 298  K. To obtain *K*_D_ values for FOXL2-DBD and all DNAs (DBE2 DNA, p53-DNA, p53-mut1 DNA and p53-mut2 DNA), 50 μM DNA prepared in a buffer containing 20 mM HEPES pH 7.5, 150 mM NaCl and 10 mM MgCl_2_ was degassed at 295  K prior to measurements. Using a microsyringe, 2.5  μl of FOXL2-DBD (750 μM) were added at intervals of 200  s to the DNA solution in the cell with gentle stirring (125 rpm). To obtain the *K*_D_ values for FOXL2-DBD and p53-DNA, 2  μl of FOXL2-DBD (850 μM) were added at intervals of 200  s to the DNA solution with 40 injections. Data were fitted to a single binding site model or multiple binding site model for p53-DNA. Data are the mean ± SEM from triplicate experiments, and the ITC thermograms are representative of triplicate experiments. Curve fitting was performed using GraphPad Prism 7.

### Size exclusion chromatography with multiangle light scattering (SEC-MALS)

SEC-MALS experiments were performed using a fast protein liquid chromatography (FPLC) system (GE Healthcare) connected to a Wyatt MiniDAWN TREOS MALS instrument and an Optilab rEX differential refractometer (Wyatt, Santa Barbara, CA, USA). A Superdex 200 10/300 GL (GE Healthcare) gel-filtration column pre-equilibrated with buffer containing 20 mM HEPES pH 7.5, 150 mM NaCl and 10 mM MgCl_2_ was used. FOXL2-DBD, FOXL2–DNA complexes, FOXA1-DBD and FOXO3-DBD proteins were injected (5–7 mg/ml) at a flow rate of 0.4 ml/min. Data were analyzed using the Zimm model for fitting static light-scattering data and graphed using the EASI graph with a UV peak in ASTRA VI (Wyatt).

### Electrophoretic mobility shift assay

DNA duplexes of p53, p53 mut1 and p53 mut2 labeled with 5′ Cy3 were purchased from Macrogen (Seoul, Korea). For p53-DNA with a 0–2 bp spacer, 5′ Cy3-labeled single-stranded DNA and complementary single strands were denatured at 95°C for 5 min and annealed at room temperature for 5 h. Next, double-stranded DNA (2.5 μM) was incubated with varying concentrations of protein. The mixtures were incubated in a reaction buffer containing 20 mM HEPES pH 7.5, 50 mM KCl, 10 mM MgCl_2_, 1 mM dithiothreitol (DTT) and 10% glycerol on ice for 30 min. For EMSA for FOXA1, a reaction buffer containing 20 mM Tris–HCl pH 8.0, 0.1 mg/ml bovine serum albumin (BSA), 50 μM ZnCl_2_, 100 mM KCl, 2 mM β-mercaptoethanol and 10% glycerol (50 mM EDTA for the Mg^2+^ depletion condition) was used (20). The reaction mixtures were loaded onto a 10% (w/v) native polyacrylamide gel and electrophoresed using 1× Tris-glycine-EDTA as the running buffer. Band intensities were quantified using ImageJ software (42).

### Cell viability assay

HeLa and SiHa cells (1 × 10^4^) were transfected using Lipofectamine 3000 for 24 h. Cell viability was measured by CellTiter-Glo Assay (Promega), according to the manufacturer's instructions.

### 5-Bromo-2′-deoxyuridine cell-proliferation assay

Proliferation of cells (1 × 10^4^) was measured using the 5-bromo-2′-deoxyuridine labeling and detection kit III (Roche, Mannheim, Germany) according to the manufacturer's instructions.

### Annexin V apoptosis assay

Apoptotic cells (1 × 10^5^) were detected using the fluoroisothiocyanate (FITC) Annexin V Apoptosis Detection Kit I (BD Bioscience, Franklin Lakes, NJ, USA) according to the manufacturer's instructions.

### FOXL2 dimer detected by *TP53* DNA pull-down

293T cells were transfected with FLAG-FOXL2 and Myc-FOXL2 expression plasmids and the indicated *TP53* promoter constructs for 24 h. Cell lysates were prepared after cross-linking by exposure to UV-C for 10 min on ice. *TP53* promoter DNA in the lysates was pulled down by biotinylated DNA probes (Probe 1: 5′-CAGGTCTTGCACCTCTTCTG-3′ and Probe 2: 5′- TACCGAGTCCCGCGGTAATT-3′), followed by purification using streptavidin beads (Invitrogen) overnight. One percent of the streptavidin beads were used for DNA extraction after incubation with proteinase K for 2 h. The extracted DNA was used to confirm that equal amounts of DNA were pulled down by real-time PCR using *TP53* promoter targeting primers (F: 5′-GCTTCTATCTTGGCGAGAAG-3′ and R: 5′-GAATGAGGGAGACAGGTCTG-3′). Proteins released by digesting DNA using DNase (Takara Bio) were immunoprecipitated with anti-FLAG, followed by SDS–PAGE and immunoblotting with the respective antibodies.

### Quantification and statistical analysis

Scatter plots were produced using GraphPad Prism (San Diego, CA, USA). The unpaired, two-tailed Student's *t*-test was used for comparisons with the control using GraphPad Prism. Values marked with letters (a, b, c, d and e) were analyzed using the Student–Newman–Keuls test, a multiple-comparison analysis, using SAS version 9.2 (SAS Institute, Cary, NC, USA). Values marked with different letters indicate significant differences. *P* < 0.05 was considered statistically significant.

## RESULTS

### Identification of *TP53* as a novel target gene of FOXL2

We found that the *TP53* promoter possesses a putative FBE by *in silico* analysis. We downloaded human ChIP-seq peak data of FOX family proteins from the ReMap database (43). ChIP-seq peak sequences from the human genome were collected and analyzed using the MEME-ChIP pipeline to discover motifs (44). We selected a motif showing a similarity to the typical FBE consensus sequence 5′-RYAAAY-3′ (R = A or G, Y = C or T) and used it to scan the *TP53* promoter region with the MAST program (45). A putative FBE was predicted at position –558 to –552 (5′-TATTTAT-3′) on the reverse strand. Using EMSA, we could confirm the direct binding of full-length FOXL2 protein with *TP53*-DNA harboring the putative FBE (Supplementary Figure S1).

**Figure 1.**
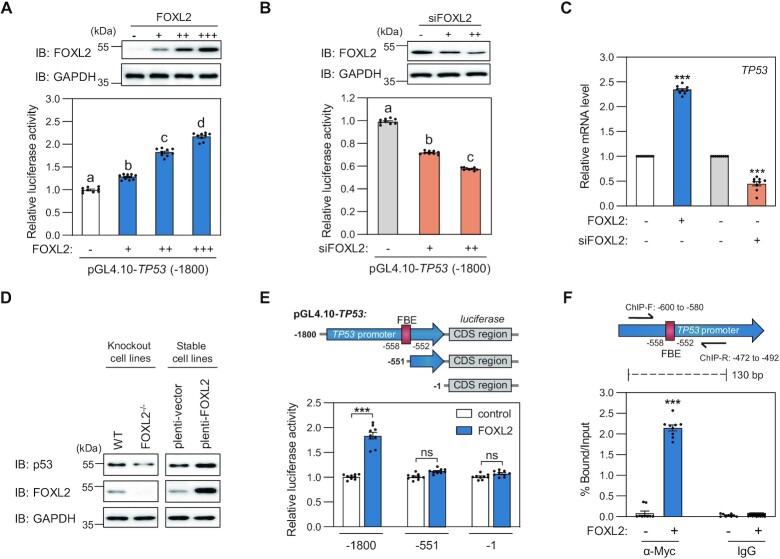
Transcriptional regulation of *TP53* by FOXL2. (**A**, **B**) Luciferase reporter assay was used to determine the activation or inhibition of *TP53* transcription in HeLa cells after transfection with increasing amounts of (A) FOXL2 expression plasmids (0, 50, 100 or 200 ng) or (B) siRNA targeting FOXL2 (0, 100 and 200 nM). Data are presented as the mean ± SEM of three independent experiments performed in triplicate. Different letters denote statistically significantly differences (*P*< 0.05). The expression of FOXL2 was confirmed by western blot. (**C**) Changes of *TP53* mRNA were analyzed in FOXL2-overexpressing or silenced HeLa cells by qRT-PCR. Data are presented as the mean ± SEM of three independent experiments performed in triplicate. Asterisks indicate statistically significant differences (***P< 0.001). (**D**) Changes of p53 protein levels in FOXL2 KO KGN cells and stably transfected KGN cells. (**E, F**) Identification of FOXL2-binding elements on *TP53* promoter DNA. pGL4.10-*TP53* promoter constructs were generated to identify the FOXL2-binding sites. (**E**) Luciferase reporter assay was performed after transfection with *TP53* constructs in HeLa cells expressing exogenous FOXL2. (**F**) ChIP assay was conducted in control or FOXL2-transfected HeLa cells using anti-Myc or IgG antibodies. The DNA region containing the FBE was amplified and quantitated by qRT-PCR using the precipitated chromatin fragment. Data are presented as the mean ± SEM of three independent experiments performed in triplicate. Asterisks indicate statistically significant differences (***P< 0.001).

To examine whether transcription of *TP53* is indeed regulated by FOXL2, we generated a reporter construct of the *TP53* promoter (–1800) and performed a luciferase reporter assay. Increased ectopic expression of FOXL2 stimulated the transcription of *TP53* (Figure 1A), whereas knockdown of FOXL2 suppressed *TP53* transcription (Figure 1B). Analogous changes in *TP53* mRNA expression upon modulation of FOXL2 levels were observed by quantitative real-time PCR (qRT-PCR) analysis (Figure 1C). Furthermore, western blot analysis indicated that FOXL2 knockout (KO) (29) or stable ectopic overexpression reduced or enhanced p53 protein levels (Figure 1D). We generated serially truncated *TP53* reporter constructs (Figure 1E). Compared with the reporter construct of the *TP53* promoter (–1800), FOXL2 failed to activate reporters that lacked the putative FBE (Figure 1E). In addition, ChIP-qPCR analysis confirmed the enrichment of *TP53* promoter sequences encompassing the FBE region in FOXL2 immunoprecipitation (Figure 1F), indicating that FOXL2 binds to the FBE of the *TP53* promoter in cells. We hypothesized that other FOX proteins might bind to the FBE region of the *TP53* promoter, considering their common binding patterns to the typical FBE (11). Indeed, other FOX proteins, including FOXA1, FOXO3, FOXD3, FOXF1, FOXI1 and FOXS1, also stimulated the transcription of *TP53* in the luciferase reporter assay (Supplementary Figure S2), suggesting that FOX proteins are previously unexplored upstream regulators of the p53 tumor suppressor.

### Crystal structure of FOXL2-DBD with p53-DNA

**Figure 2.**
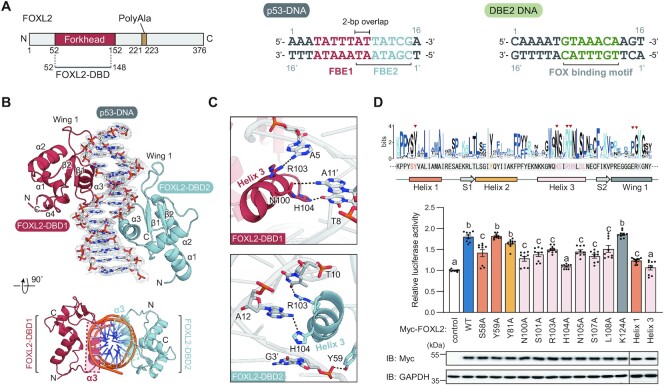
Crystal structure of FOXL2 in complex with p53-DNA. (**A**) Domain architecture of FOXL2 and DNA sequences used in this study. The construct used in this study is indicated by a gray line. The two DNA sequences (p53-DNA and DBE2 DNA) used in the structural study are shown. The FOX-binding sites observed in the crystal structures are highlighted in color. (**B**) Crystal structure of the FOXL2-DBD in complex with p53-DNA. DNA sequences are shown, and FOXL2-DBD1 and FOXL2-DBD2 are colored in red and skyblue, respectively. The picture at the bottom shows a 90° rotation around the horizontal axis, and helix 3 in each DBD is highlighted in a dashed box. 2Fo-Fc electron density maps (1.0 σ) of DNA are shown. (**C**) Magnified views show details of the interaction at the interface of p53-DNA and FOXL2-DBD1 or FOXL2-DBD2. Hydrogen bonds are represented by black dashed lines. (**D**) The effect of mutations of FOXL2 key binding residues on *TP53* transcriptional activation was determined by luciferase reporter assay. Data are presented as the mean ± SEM of three independent experiments performed in triplicate. Different letters denote statistically significantly differences (*P*< 0.05). The equal expression of FOXL2 mutants was detected by western blot, and GAPDH was used as a loading control.

To gain structural insight into how FOX proteins bind to the *TP53* promoter, we determined the co-crystal structure of FOXL2-DBD in complex with the p53-DNA (5′-AAATATTTATTATCGA-3′) (Figure 2A–C). Unexpectedly, two FOXL2-DBDs (FOXL2-DBD1 and FOXL2-DBD2) were found to bind to p53-DNA with a 2:1 stoichiometry in the crystal structure (Figure 2B). FOXL2-DBD1 binds to the FBE (5′-TATTTAT-3′, hereafter FBE1) in the major groove of p53-DNA through the α3-helix, whereas FOXL2-DBD2 binds to another major groove (5′-ATTATCG-3′, hereafter FBE2) adjacent to the FBE1 site, with two overlapping bases. Both FOXL2-DBD1 and FOXL2-DBD2 showed a typical winged-helix structure, consisting of five α-helices, two antiparallel β-strands and two characteristic flexible loops, which are referred to as wings 1 and 2 (Figure 2B). Since the complementary sequence of the FBE1 site (5′-ATAAATA-3′) belongs to the typical FBE 5′-RYAAAYA-3′ (R = A or G, Y = C or T), the binding mode of FOXL2 to DNA was distinct from the crystal structures of other FOX proteins, i.e. the direction of FOXL2 was opposite to that of other structurally characterized FOX structures, with wing 1 heading to the 5′ site of p53-DNA (Figure 2B; Supplementary Figure S3). In FOXL2-DBD1, His104 made direct contact with the base of T8, and the N atom of Asn100 interacted with the base of A11′ (2.3–3.5 Å). The N atom of Arg103 formed a network with the base of A5 (3.0 Å) (Figure 2C, top). Similar binding patterns were observed for FOXL2-DBD2 (Figure 2C, bottom). His104 interacted with the base of A12 (3.0 Å), whereas Arg103 interacted with the base of T10 (2.3 Å). In addition, Tyr59 interacted with the phosphate backbone of G3′ (2.5 Å) (Figure 2C, bottom).

To further demonstrate that key residues of FOXL2 protein that were identified from structural analyses are functionally relevant for *TP53* transcription activation, critical FOXL2 residues involved in interaction with *TP53* promoter DNA were mutated to alanine, and a luciferase reporter assay was performed. Compared with the FOXL2 wild type, FOXL2 mutants (S58A, N100A, S101A, R103A, H104A, N105A, S107A and L108A) exhibited lower transcriptional activity on the *TP53* promoter, whereas Y59A, Y81A and K124A mutants showed no significant change in their transcriptional activities on *TP53* (Figure 2D). In addition, FOXL2 with multiple mutations in helix 1 (S58A and Y59A) or helix 3 (N100A, S101A, R103A, H104A, N105A, S107A and L108A) showed significantly compromised transcriptional activation of *TP53* (Figure 2D).

### Crystal structure of FOXL2-DBD with DBE2 DNA

**Figure 3.**
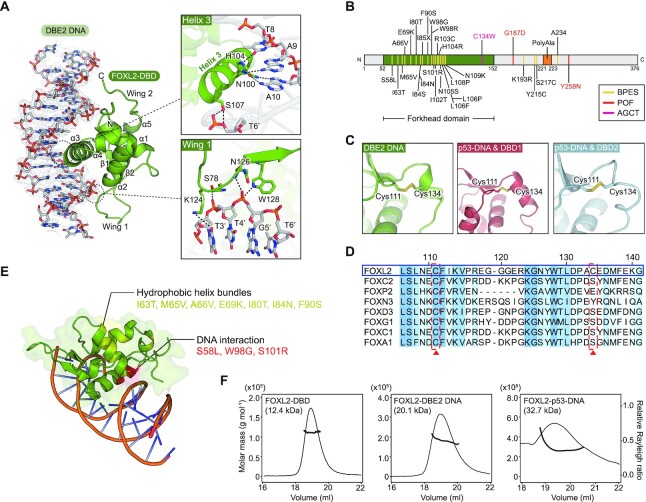
Disease-associated mutations in FOXL2. (**A**) Crystal structure of FOXL2-DBD in complex with DBE2 DNA. The DNA sequences used are shown, and FOXL2-DBD is colored in green. 2Fo-Fc electron density maps (1.0 σ) of DNA are shown. On the right side, magnified views show details of the interaction at the interface of DBE2 DNA and FOXL2-DBD (helix 3 and wing 1). Hydrogen bonds are represented with black dashed lines. (**B**) Representative disease-associated mutations in FOXL2 are indicated in domain architecture. The forkhead domain is colored in green. Mutation sites related to BPES, POF and adult granulosa cell tumor (AGCT) are colored in yellow, orange and pink, respectively. (**C**) Disulfide bond observed in crystal structures of FOXL2. Cys111 and Cys134 in each FOXL2-DBD (FOXL2-DBD in the DBE2 DNA complex, FOXL2-DBD1 and FOXL2-DBD2 in the p53 DNA complex) are shown in green, red and skyblue, respectively. (**D**) Sequence alignment of FOX proteins is shown. Conserved residues are colored in blue. Cys111 and Cys134 are highlighted in a red box. (**E**) BPES-associated mutations are shown in the crystal structure of FOXL2, and classified into two groups; hydrophobic helix bundles in yellow and DNA interaction in red. (**F**) The molecular weight of FOXL2-DBD, FOXL2-DBD in complex with DBE2 DNA and FOXL2-DBD in complex with p53-DNA was measured by SEC-MALS. The thick line represents measured molecular mass.

To compare the binding mode of FOXL2-DBD to the *TP53* promoter with that of typical FBEs and reveal the unique binding mode of FOXL2-DBD, we determined the co-crystal structure of FOXL2-DBD in complex with 16 bp DNA (5′-CAAAATGTAAACAAGT-3′, hereafter DBE2 DNA) containing DBE2 with GTAAACA as a consensus motif (Figures 2A and 3A). FOXL2-DBD bound to the DBE2 DNA with 1:1 stoichiometry in the asymmetric unit, which was similar to previous structures of other FOX proteins and the DBE2 DNA complex with root mean square deviations (RMSDs) for all Cα atoms of ∼0.33–0.51 Å (Supplementary Figure S3) (46–51). Interestingly, the stoichiometry between FOXL2 and DNA was distinct depending on the DNA sequence (p53-DNA or DBE2 DNA).

Similar to the FOXL2:p53-DNA complex, the α3 helix in FOXL2 directly contacts the consensus motif (5′-GTAAACA-3′) in the major groove of DBE2 DNA. Highly conserved residues (Asn100, His104 and Ser107) play a role as key interaction residues with DNA. Asn100 formed a bidentate interaction with the base of A10 (3.1 and 3.4 Å, respectively), while His104 was mainly inserted into the major groove in DNA and formed hydrogen bonds with T8 and T9. In addition, Ser107 bound to the phosphate backbone of T6′ (2.6–2.7 Å) (Figure 3A, top). The wing 1 region contributed to additional contacts between FOXL2 and the backbone of the DBE2 DNA. Asn126 and Trp128 bound to the phosphate backbone in G5′. Ser78 bound to the T4′ backbone. Notably, the side chain of Lys124 was deeply inserted and bound to the base of T3′ toward the minor groove of DBE2 DNA (Figure 3A, bottom). Due to the flexibility of the C-terminus, 140–158 residues in wing 2 are not shown in the electron density map.

Given the contributions of residues at the interface between FOXL2 and the two DNAs, most disease-related mutations are clustered in the forkhead domain (Figure 3B, C). Among them, the C134W mutation causes adult-type granulosa cell tumors. Notably, Cys111 is a highly conserved residue in FOX proteins, but Cys134 is a distinct characteristic of FOXL2 (Figure 3D, E). Cys134 formed a disulfide bond with Cys111, connecting wings 1 and 2, thereby decreasing the flexibility of wing 2 and bringing wing 2 near the minor groove of DNA, suggesting that disruption of the disulfide bond would be related to adult-type granulosa cell tumors (Figure 3C).

To verify the stoichiometry observed in the crystal structure, we measured the molecular weight in solution by SEC-MALS. The molecular weight of FOXL2-DBD was 12.4 kDa, indicating the monomeric state of FOXL2-DBD (Figure 3F), while those of FOXL2-DBDs in complex with DBE2 DNA or p53-DNA were 20.1 and 32.7 kDa, respectively, which showed consistent 1:1 and 2:1 stoichiometry in solution, similar to the crystal structure (Figure 3F). The binding of FOXL2-DBD to p53-DNA with a 2:1 stoichiometry is unique to the *TP53* promoter sequence.

### Cooperativity analysis of FOXL2 and the *TP53* promoter

**Figure 4.**
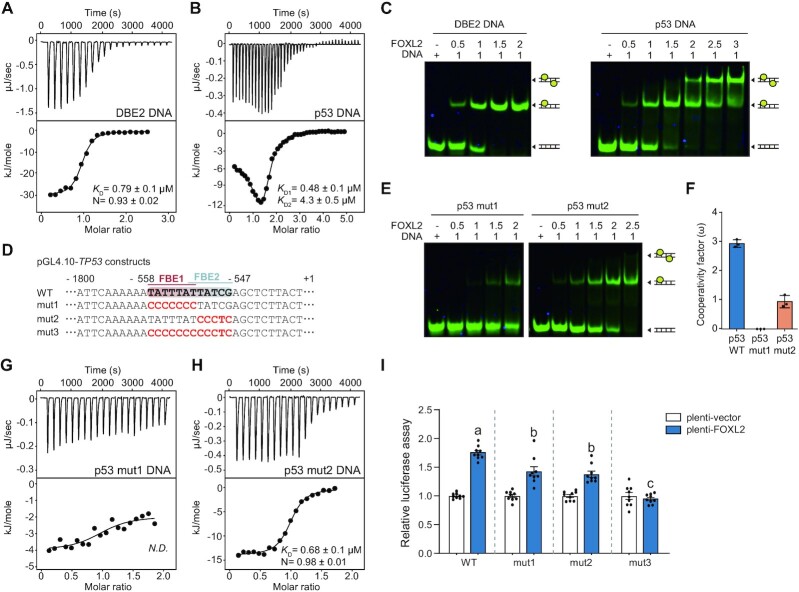
Cooperative binding pattern of FOXL2 on the *TP53* promoter. (**A**,**B**) Representative ITC fitting results of FOXL2-DBD with (A) DBE2 DNA and (B) p53-DNA. The thermodynamic data were collected by titrating FOXL2-DBD into each DNA, and the parameters were calculated by fitting to a single-binding model for DBE2 DNA or multiple-site binding model for p53-DNA. (**C**) EMSA results of FOXL2-DBD using DNA probes with DBE2 DNA and p53-DNA. Cy5-labeled double-stranded DNA (2.5 μM) was incubated at the indicated molar ratio. The dimer, monomer and free DNA are illustrated. (**D**) Mutant DNA sequences in pGL4.10-TP53 constructs. FBE1 and FBE2 sites are colored in red and skyblue, respectively. Mutated sequences are highlighted in red. (**E**) EMSA results of FOXL2-DBD using DNA probes with p53 mut1 DNA and p53 mut2 DNA. Cy5-labeled double-stranded DNA (2.5 μM) was incubated at the indicated molar ratio. (**F**) Cooperativity factors (ω) calculated from the fractions of band intensity observed in the quantitative EMSAs (52). The data are presented as means ± SEM (*n* = 3). Relative fraction intensities are shown in Supplementary Figure S4. (**G**,**H**) Representative ITC fitting results of FOXL2-DBD with (G) p53 mut1 DNA and (H) p53 mut2 DNA. The thermodynamic data were collected by titrating FOXL2-DBD into each DNA, and the parameters were calculated by fitting to a single-binding model. (**I**) Luciferase reporter assay with KGN cells stably expressing FOXL2 using the wild type (WT), mut1 and mut2 pGL4.10-*TP53* constructs. Data are presented as the mean ± SEM of three independent experiments performed in triplicate. Different letters denote statistically significantly differences (*P*< 0.05).

To investigate the binding mode of FOXL2 molecules in the *TP53* promoter and DBE2 DNA, we measured binding affinity and stoichiometry using ITC. FOXL2-DBD bound to the DBE2 DNA with an apparent *K*_D_ of 0.79 ± 0.1 μM and with 1:1 stoichiometry, which is consistent with its crystal structure. Consistent with the crystal structure, FOXL2-DBD bound to p53-DNA with 2:1 stoichiometry. Surprisingly, it showed a biphasic binding pattern with heat changes (Figure 4A, B), suggesting that there might be cooperative binding of two FOXL2 molecules with p53-DNA.

To further verify the cooperativity of FOXL2 in the *TP53* promoter, we performed an EMSA. When the 5′-Cy3-labeled DBE2 DNA was incubated with excess molar FOXL2-DBD, a monomeric band shift was observed in response to the gradient protein concentration of FOXL2-DBD, consistent with the ITC results (Figure 4C). In 5′-Cy3-labeled p53-DNA, a monomeric band shift was observed when FOXL2-DBD was incubated with the same molar ratio with the DNA, but a dimeric band shift was additionally observed when excess FOXL2-DBD was added to DNA (Figure 4C). To quantify these band shifts, we calculated the cooperativity factor (ω), which indicates cooperative patterns in protein–DNA interactions (52) (Figure 4F). Briefly, ω > 1 indicates that proteins bind to DNA in a positive cooperativity manner, ω = 1 indicates no cooperativity and ω < 1 indicates negative cooperativity. The calculated cooperativity factor for p53 wild-type DNA was 2.96 ± 0.45, which indicates positive cooperativity (Figure 4F; Supplementary Figure S4A). These data suggest that either FOXL2-DBD1 or FOXL2-DBD2 has a positive cooperative effect on the binding of the other molecule.

To test this hypothesis, we designed three mutants of p53-DNA, p53 mut1, p53 mut2 and p53 mut3, which replace the FOXL2-DBD1-binding sequence FBE1 with 5′-CCCCCC-3′, the FOXL2-DBD2-binding sequence FBE2 with 5′-CCCTC-3′ and both sequences with 5′-CCCCCC-3′ and 5′-CCCTC-3′, respectively (Figure 4D). Using p53 mut1 DNA, the dimer band shift disappeared, and only a weak monomer shift was observed (Figure 4E). In p53 mut2 DNA, the dimer band shift was weakened compared with wild-type p53-DNA (Figure 4E). In addition, for p53 mut2 DNA, the cooperativity factor was 0.96 ± 0.18, which indicates that the cooperativity mostly vanished (Figure 4F; Supplementary Figure S4B, C). Consistently, the ITC biphasic pattern of FOXL2 binding to wild-type p53-DNA disappeared, and instead a 1:1 one-site binding curve was observed when FOXL2-DBD was titrated into p53 mut2 DNA solution. Notably, the binding of FOXL2-DBD to p53 mut1 DNA was greatly weakened (Figure 4G, H). These results suggest that FOXL2-DBD1 binds to the FBE1 site first and, subsequently, the minor groove width becomes narrower to create the new binding site FBE2 by increasing its binding affinity via DNA allostery. Next, FOXL2-DBD2 binds to the FBE2 site. To further determine whether the cooperative binding of FOXL2 to p53-DNA identified by structural analyses is a biologically significant event, we performed luciferase reporter assays in the FOXL2-stable cell line using the three mutants of the *TP53* promoter mentioned above, mut1, mut2 and mut3 (Figure 4D, I). Compared with the magnitude of the wild-type *TP53* promoter reporter activation, lower transcriptional activation of p53 mut1 and mut2 promoter constructs was observed, whereas no transcriptional activation was observed for mut3, which lacks both FBE1 and FBE2 sequences (Figure 4I). Taken together, these results suggest that FOXL2-DBD1 binds first to the FBE1 site, and subsequently FOXL2-DBD2 binds to the FBE2 site with increased binding affinity, indicating that FOXL2-DBD1 and FOXL2-DBD2 positively cooperate for binding to the *TP53* promoter.

### Cooperative binding of other FOX proteins to the *TP53* promoter

**Figure 5.**
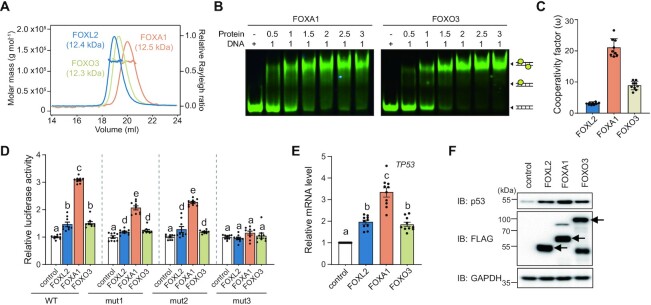
Cooperative binding pattern of FOX proteins on the *TP53* promoter. (**A**) The molecular weight of FOXL2, FOXO3 and FOXA1 measured by SEC-MALS. FOXL2, FOXO3 and FOXA1 are colored in blue, beige and orange, respectively. The thick line represents the measured molecular mass. (**B**) EMSA results of FOXA1-DBD and FOXO3-DBD using p53-DNA probes. Cy5-labeled double-stranded DNA (2.5 μM) was incubated at the indicated molar ratio. The dimeric protein–DNA complex, monomeric protein–DNA complex and free DNA are illustrated. (**C**) Cooperativity factors (ω) calculated from the fractions of band intensity observed in the quantitative EMSAs. FOXL2, FOXO3 and FOXA1 are colored in blue, beige and orange, respectively. Relative fraction intensities are shown in Supplementary Figure S4D and E. Data are presented as the mean ± SEM of three independent experiments performed in triplicate. (**D–F**) The regulation of *TP53* transcription by FOX proteins (FOXO3 and FOXA1) was detected by (D) luciferase assay using wild-type (WT), mut1 and mut2 pGL4.10-TP53 constructs, (E) *TP53* mRNA levels, (F) p53 protein levels. Data are presented as the mean ± SEM of three independent experiments performed in triplicate. Different letters denote statistically significantly differences (*P*< 0.05).

Given that the forkhead domains are highly conserved in various FOX proteins and their transcriptional activities on the *TP53* promoter are also conserved (Supplementary Figures S2 and S5), we anticipated that other FOX proteins would show similar binding patterns with the *TP53* promoter. We chose FOXA1 and FOXO3 proteins for further studies. Notably, FOXA1 interacts with the *TP53* promoter (53). SEC-MALS experiments with purified forkhead domains of FOXA1 and FOXO3 indicated that both proteins were monomeric in solution (FOXA1-DBD, 12.5 kDa; and FOXO3-DBD, 12.3 kDa) (Figure 5A).

Next, we performed EMSA experiments to examine whether FOXA1 and FOXO3 proteins cooperatively bind to the *TP53* promoter, similarly to FOXL2. Indeed, dimeric band shifts were observed when excess levels of these proteins were added gradually to p53-DNA (Figure 5B). Given that the calculated cooperativity factors of FOXA1 and FOXO3 were 21.02 ± 2.88 and 8.87 ± 1.37, respectively, we concluded that both FOXA1 and FOXO3 also bind to the *TP53* promoter cooperatively (Figure 5C; Supplementary Figure S4D, E). Since, similarly to FOXL2, FOXA1 and FOXO3 showed cooperative binding to the *TP53* promoter, we performed parallel luciferase reporter assays in the FOXL2-stable cell line. As presented in Figure 5D, FOXO3 exhibited transcriptional activity toward *TP53* to a similar extent as FOXL2, whereas FOXA1 showed much stronger activation of *TP53* transcription. When p53 mut1, mut2 or mut3 promoters were tested in cells overexpressing FOXA1 or FOXO3, we obtained results consistent with those of FOXL2: lower transcriptional activation with p53 mut1 or mut2 and no transcriptional activity with p53 mut3 (Figures 4I and 5D). Similar levels of up-regulation of *TP53* mRNA expression in cells expressing exogenous FOXL2, FOXA1 or FOXO3 were confirmed by qRT-PCR analysis (Figure 5E). These increased *TP53* mRNA levels led to the up-regulation of p53 protein expression, where FOXA1 exhibited higher activity (Figure 5F). Taken together, these results suggest that the cooperative binding patterns of FOX proteins to the *TP53* promoter might be a general mechanism for regulating p53 expression by the FOX family of transcription factors.

### Crystal structure of FOXA1-DBD with p53-DNA

**Figure 6.**
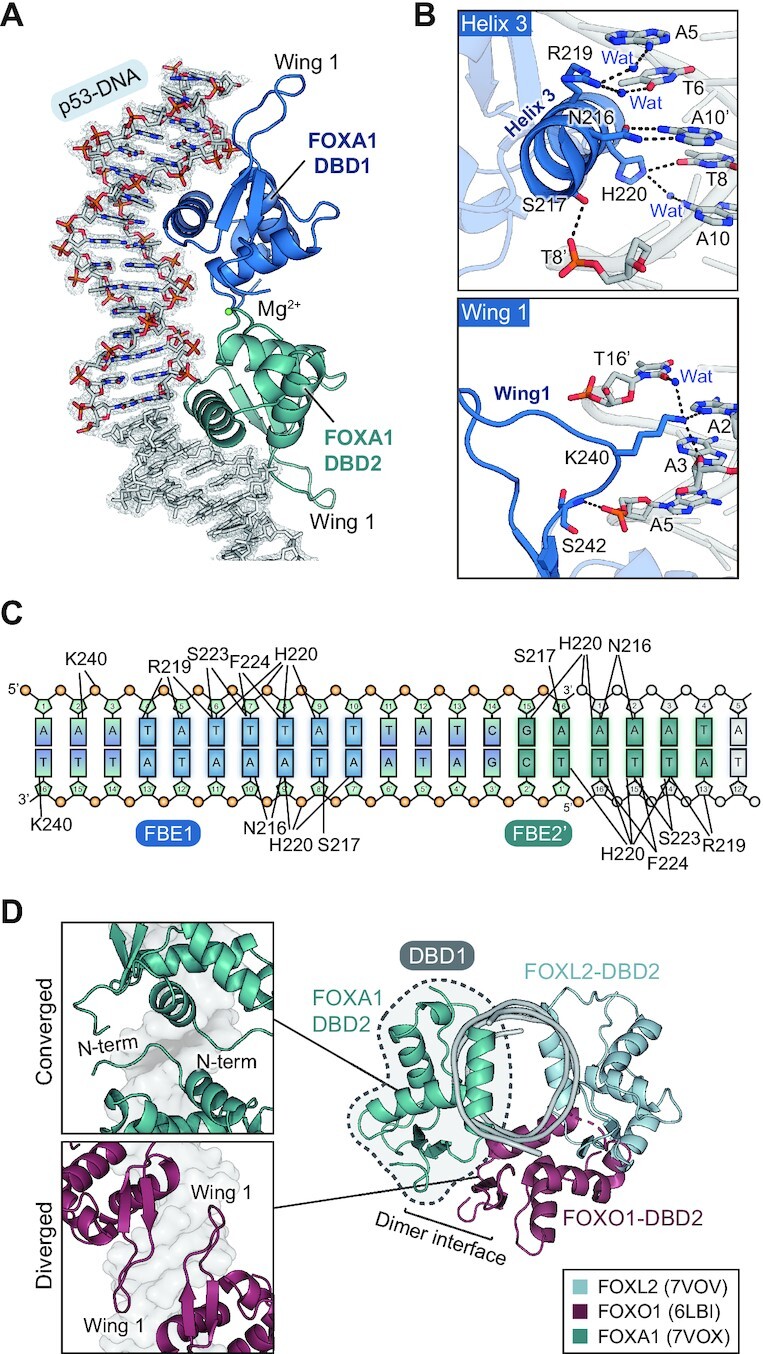
Crystal structure of FOXA1 in complex with p53-DNA. (**A**) Overall crystal structure of FOXA1-DBD in complex with p53-DNA. DNA sequences are shown. FOXA1-DBD1 and FOXA1-DBD2 are colored in blue and light teal, respectively. 2Fo-2c electron density maps (1.0 σ) of DNA are shown. (**B**) Magnified views showing details of the interaction at the interface of p53-DNA with FOXA1-DBD1 (helix 3 and wing 1). Hydrogen bonds are represented with black dashed lines. Water molecules are shown in blue spheres. (**C**) Schematic diagram of the FOXA1-DBD and p53-DNA interactions observed in the crystal structure. (**D**) Structural comparisons of the FOXL2-DBD:p53-DNA complex, FOXA1-DBD:p53-DNA complex and FOXO1-DBD in complex with DIV2 DNA (PDB ID: 6LBI). The location of FOX-DBD1 molecules is shown in a dashed circle. The dimer interface between FOXA1-DBD or FOXO1-DBD molecules is shown in magnified view. FOXL2-DBD, FOXA1-DBD and FOXO1-DBD are colored in skyblue, light teal and burgundy, respectively.

We further determined the co-crystal structure of FOXA1-DBD in complex with p53-DNA (5′-AAATATTTATTATCGA-3′). The crystals of the FOXA1-DBD complex with p53-DNA also showed 2:1 stoichiometry, and FOXA1-DBD1 bound to the major groove of the FBE1 site through helix 3 of FOXA1-DBD, which is similar to FOXL2-DBD (Figure 6A). However, FOXA1-DBD2 bound to DNA at an unexpected position distinct from FOXL2, where helix 3 of FOXA1-DBD2 bound to the major groove of extended DNA formed by crystal packing, and formed apparent protein–protein dimerization with FOXA1-DBD1 (Figure 6A). In FOXA1-DBD1, His220 (corresponding to His104 in FOXL2) made contact with the base of T8′ and also with that of A10 via a water molecule (3.0–3.3 Å). Asn216 (Asn100 in FOXL2) formed bidentate hydrogen bonds with the base of A10′ (2.9–3.1 Å) (Figure 6B, C). Arg219 formed two water-mediated contacts with the bases A5 and T6. Ser217 made additional contacts with the phosphate backbone of T8′. In the wing 1 region, the backbone of Ser242 and that of A5 formed a hydrogen bond, and the side chain of Lys240 was directed to the major groove of DNA, interacting with the bases of A2 and A3, and water-mediated interaction with T16′ (3.3 Å), constituting additional contacts between FOXA1 and p53-DNA (Figure 6B, C).

### Structural insights into the cooperative binding of FOX proteins to the *TP53* promoter

**Figure 7.**
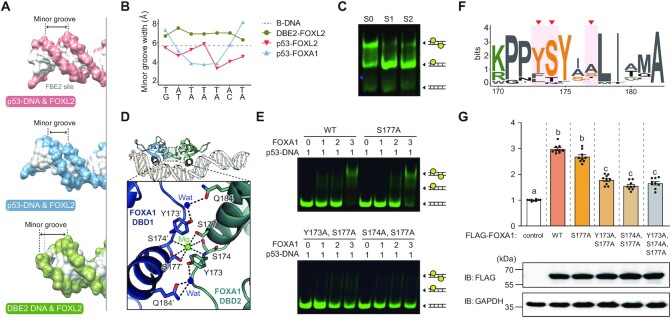
DNA allostery is a key factor for homotypic cooperativity of FOX proteins. (**A**) DNA surfaces in three crystal structures of FOX-DNA complexes (FOXL2-DBD:DBE2 DNA, FOXL2-DBD:p53-DNA and FOXA1-DBD:p53-DNA in green, red and skyblue, respectively). Black arrows indicate the minor groove width. (**B**) Minor groove widths of DNA in the structures of B-DNA (gray), FOXL2-DBD:DBE2 DNA (green), FOXL2-DBD:p53-DNA (red) and FOXA1-DBD:p53-DNA (skyblue). Groove paramaters were analyzed using Curves+ (83). (**C**) EMSA results of FOXL2-DBD using the probes p53-DNA (S0), 1 bp spacer p53-DNA (S1) and 2 bp spacer p53-DNA (S2). The dimeric protein–DNA complex, monomeric protein–DNA complex and free DNA are illustrated. For spacer design, see also Supplementary Figure S6B. (**D**) Details of the interactions at the interface between FOXA1-DBD1 (blue) and FOXL2-DBD2 (light teal). Hydrogen bonds are represented by black dashed lines. Water molecules and Mg^2+^ are shown in blue and green spheres, respectively. (**E**) EMSA results of FOXA1 variants, which substituted the key interaction residues to alanine, using p53-DNA probes. FOXA1 variants of S177A, Y173A/S177A and S174A/S177A mutation were used. The dimeric protein–DNA complex, monomeric protein–DNA complex and free DNA are illustrated. (**F**) Sequence logos of the corresponding region encompassing Y173, S174 and S177 of FOXA1 among FOX family proteins. The positions of Y173, S174 and S177 in FOXA1 are highlighted with red triangles. (**G**) Results of the luciferase reporter assay performed in cells transfected with plasmids encoding different FOXA1 mutants (S177A, Y173A/S177A, S174A/S177A and Y173A/S174A/S177A). Equal expression of FOXA1 mutants was determined by western blot. GAPDH was used as a loading control. Data are presented as the mean ± SEM of three independent experiments performed in triplicate. Different letters denote statistically significantly differences (*P*< 0.05).

We next investigated the structural determinants of the cooperative binding of FOX proteins to the *TP53* promoter. To examine the structural differences in p53-DNA and DBE2 DNA backbones that mediate binding to FOX proteins, we aligned three DNA backbone structures solved in this study and analyzed their groove parameters (Figure 7A, B). Surprisingly, the overall minor groove width in p53-DNAs was distinctly smaller than that in standard right-handed B-DNA and DBE2 DNA (3.4–5.9 Å) (Figure 7B; Supplementary Figure S6A). In comparison, the overall minor groove width in the DBE2 DNA was larger than that in B-DNA (6.3–7.5 Å and 5.7 Å for DBE2 DNA and B-DNA, respectively).

Based on these structural differences in groove parameters, we hypothesized that when FOXL2-DBD1 binds to the FBE1 site, the minor groove width might become narrower to create the new binding site FBE2 for the binding of FOXL2-DBD2, which means a DNA-mediated allostery. To further investigate whether the subsequent binding of FOXL2-DBD2 to the FBE2 site is DNA sequence specific, we inserted a 1 or 2 bp spacer between FBE1 (5′-TATTTAT-3′) and FBE2 sites (5′-TATCG-3′) (Figure 7C; Supplementary Figure S6B). Similarly, the effect of altered half-site spacing on FOXO1 cooperativity was tested using various spacers (DIV0–DIV4) in a previous study (17). Since p53 wild-type DNA possesses two binding sites with two overlapping bases, which is similar to DIV0 DNA, we inserted a 1 or 2 bp spacer so that the spacing of p53-DNA resembles that of DIV1 or DIV2 DNA, respectively. Compared with p53 wild-type DNA (S0), the dimeric band shift was significantly reduced when a 1 or 2 bp spacer (S1 and S2, respectively) was inserted into the p53-DNA (Figure 7C; Supplementary Figure S6B). These reduced dimeric shifts indicate that the subsequent binding of FOXL2-DBD2 was not dependent on the DNA sequence of the FBE2 site, but the DNA allostery-induced position of the FBE2 site is crucial. Thus, we concluded that DNA allostery largely contributes to the positive cooperativity of FOXL2 binding to p53-DNA. However, it should be noted that the p53 mut1 weakly binds to FOXL2 (Figure 4E), which implies that binding of FOXL2 to the FBE2 site does not exclusively rely on the DNA groove allostery, but FBE2 sequence specificity also contributes to the binding to a certain extent.

### Homodimerization further increases cooperative binding of FOXA1 on the *TP53* promoter

Distinct from FOXL2, the protein dimer interface between the N- and C-termini of FOXA1-DBD1 and FOXA1-DBD2 was observed in the structure of FOXA1-DBD in complex with p53-DNA (Figure 7D). Tyr173 interacted with Gln184 in neighboring FOXA1-DBD molecules. Importantly, Ser174 and Ser177 in FOXA1-DBD1 and FOXA1-DBD2 formed Mg^2+^ coordination with their backbone and side chains (Figure 7D). To confirm whether the dimer interface is actually formed in solution and is not a crystallographic artifact, we conducted EMSA under Mg^2+^ depletion conditions. Indeed, the dimeric band shift was significantly reduced following treatment with excess EDTA, indicating that the Mg^2+^-mediated dimer interface actually affects the cooperativity (Figure 7E; Supplementary Figure S6C). In addition, when the N- and C-termini harboring key interacting residues in the dimer interface were truncated, relative luciferase activities of all truncated FOXA1 proteins (ΔFH-N, ΔFH-N and ΔFH-N&C) on the *TP53* promoter were decreased by two-thirds compared with that of wild-type FOXA1 (Supplementary Figure S6D, E). In more detail, the key interfacial residues in FOXA1 (S177A, Y173A/S177A and S174A/S177A) were substituted to alanine, and changes in the dimeric band shift were observed by EMSA. While the S177A mutant showed little change to *TP53* binding, double mutants of either Y173A/S177A or S174A/S177A exhibited a significantly compromised interaction (Figure 7E). In addition, single and double mutants of FOXA1 expression plasmids were generated, and luciferase assays for *TP53* activation were performed. Consistent with the EMSA result (Figure 7E), the S177A mutant of FOXA1 exhibited little reduction whereas the double mutants (Y173A/S177A and S174A/S177A) of FOXA1 showed significantly reduced luciferase activities to a similar extent to that observed with the triple mutant (Y173A/S174A/S177A) of FOXA1 (Figure 7G). The remaining luciferase activities of truncated or alanine-substituted FOXA1 mutants were similar to those of wild-type FOXL2 and FOXO3 (Figure 5D), suggesting that DNA allostery also contributes to the binding of FOXA1-DBD2 to the FBE2 site. Overall, these results indicate that the dimerization of FOXA1 on the *TP53* promoter might be an additional factor for the stronger cooperative binding of FOXA1 to the *TP53* promoter compared with other FOX proteins.

### Biological roles of *TP53* regulation by FOX proteins in cancer

**Figure 8.**
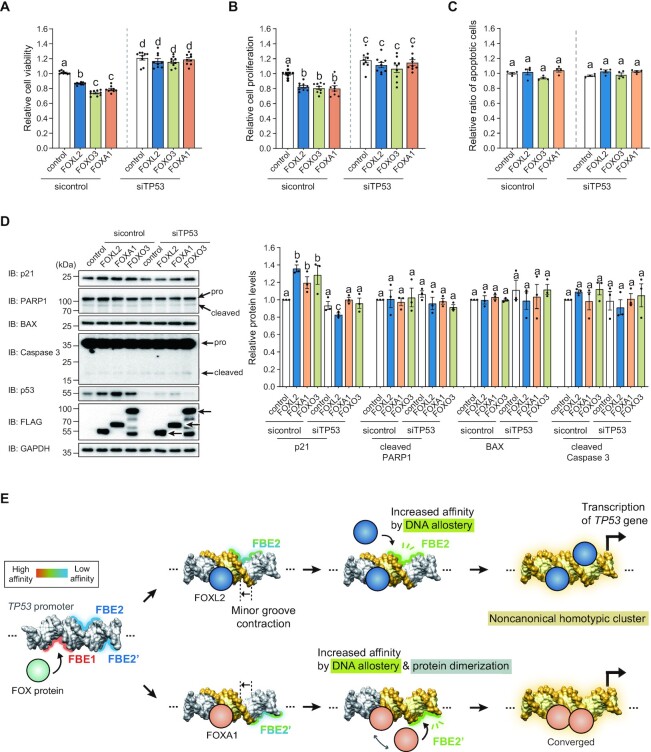
Homotypic cooperativity of FOX proteins in modulating the *TP53* promoter. The effect of FOX proteins on (**A**) cell viability, (**B**) proliferation and (**C**) apoptosis was examined in control or siTP53-silenced HeLa cells. Data are presented as the mean ± SEM of three independent experiments performed in triplicate. Different letters denote statistically significant differences (*P*< 0.05). (**D**) Protein changes involving the cell cycle (p21 level) and apoptosis (BAX level and cleaved PARP1 and Caspase 3) in HeLa cells were assessed by western blot analysis using the respective antibodies. Representative blots (left panel) and quantitative data (mean ± SEM; right panel) from three independent experiments performed are presented. (**E**) Proposed model of DNA allostery mechanism of FOX proteins in regulation of the *TP53* homotypic cluster. The minor groove width became narrower when FOX proteins bound to the FBE1 site, creating the second binding site FBE2 (or FBE2′) by increasing their binding affinities.

Because FOXL2, FOXO3 and FOXA1 exhibit tumor-suppressive functions (27,54,55) and p53 is the crucial tumor suppressor controlling the cell cycle, apoptosis, metastasis and differentiation (56), the biological role of transcriptionally activated *TP53* was investigated. We assessed the viability of HeLa cells, a cervical carcinoma cell line, in which p53 is down-regulated by the human papillomavirus E6 protein through ubiquitin-mediated degradation (57). Ectopic expression of FOXL2, FOXO3 and FOXA1 proteins resulted in reduced cell viability, but FOX proteins failed to decrease cell viability when *TP53* was silenced in the cells (Figure 8A). Next, we examined whether this decrease in cancer cell viability was due to inhibition of cell proliferation or an increase in apoptotic cell death. As shown in Figure 8B, FOX proteins significantly inhibited HeLa cell proliferation, and these inhibitory activities were not observed in *TP53-*silenced HeLa cells. In contrast, FOX proteins did not show a significant effect on the apoptotic death of HeLa cells and *TP53-*silenced HeLa cells (Figure 8C). We observed consistent results from another cervical carcinoma cell line, SiHa (Supplementary Figure S7A–C). Furthermore, molecular changes involving the cell cycle and apoptosis were assessed by western blot analysis. As shown in Figure 8D, FOXL2, FOXO3 and FOXA1 proteins promoted expression of p21 that arrests the cell cycle, while this p21 up-regulation was not observed in *TP53* knockdown cells. In contrast, ectopic expression of FOX proteins did not significantly affect BAX expression and cleavages of PARP1 and Caspase 3 in both control and *TP53* knockdown cells (Figure 8D). These results suggest that FOX-induced anti-proliferative activity in these cancer cells is mediated by *TP53* up-regulation.

## DISCUSSION

The tumor suppressor protein p53 is a major cellular ‘gatekeeper’, which inhibits various steps of tumor development. Thus, precise control of *TP53* transcription by upstream regulators is crucial. In this study, we revealed that FOX proteins comprising a large family of transcription factors in the human genome are upstream regulators of p53, showing that FOX proteins, including FOXL2, FOXA1, FOXO3, FOXD3, FOXF1, FOXI1 and FOXS1, stimulated the transcription of *TP53* (Supplementary Figure S2). Based on these observations, it is tempting to hypothesize that binding of FOX proteins to the *TP53* promoter is a broadly conserved event. Previous studies have consistently reported a potential cross-talk between p53 and FOXO from the perspective of cell cycle progression in response to cellular stress (58,59). In addition, FOXA1 and FOXD3 were shown to up-regulate the protein levels of p53 in colorectal cancer cells (53,60). Nevertheless, identification and mapping of *TP53* promoter sites where FOX transcription factors bind are largely unexplored. Here, we identified a novel and a common binding site for FOX family transcription factors on the *TP53* promoter, and structural studies revealed that FOX proteins bind to the *TP53* promoter in a 2:1 stoichiometry, which demonstrates the existence of homotypic clusters.

Homotypic clusters of transcription factors (HCTs), which are clusters of multiple adjacent binding sites for the same transcription factors, are a complex system of regulation of promoters or other *cis*-regulatory elements for gene expression in vertebrate genomes (61–64). Analysis of evolutionarily conserved HCTs showed that the promoters of transcription factors possess highly enriched HCTs, and binding of transcription factors in these clusters, which occupy nearly 2% of the human genome, enables diverse but exquisite regulation of gene expression (62–65). The effect of a HCT on gene expression depends on how binding of one molecule of a transcription factor influences that of another molecule, either independently or cooperatively (66). In this study, biophysical assays, such as ITC and EMSA, implicated the cooperative binding of FOX proteins to the HCT of the *TP53* promoter. *In vivo* cross-linking experiments also showed that the formation of FOXL2 dimer on the *TP53* promoter depends on both FBE1 and FBE2 sites of the *TP53* promoter. Dimeric FOXL2 in mutant *TP53*-transfected cells was significantly lower than that in wild-type *TP53* promoter-transfected cells (Supplementary Figure S6F).

Regarding the cooperative mechanism of interaction of transcription factors with a HCT, two possibilities exist: either protein-mediated or DNA-mediated cooperativity. In our study, except for FOXA1, there was no direct interaction between the two FOX-DBD molecules in the crystal structures. Importantly, all determined crystal structures of FOX proteins (FOXL2 and FOXA1) in complex with p53-DNA exhibited a significant change in DNA shape. The minor groove width became narrower when FOXL2 and FOXA1 bound to the FBE1 site, creating the second binding site FBE2 by increasing their binding affinities (Figure 8E). These results demonstrate the important role of exquisite DNA allostery in the binding of the second molecule of the transcription factor in a positive cooperative manner (67,68).

Previous structural studies about homodimerization of FOX proteins can be classified into two groups: DNA-mediated homodimer with protein–protein interaction (PPI) or DNA-mediated homodimer without PPI (17,19,20,38) (Supplementary Figure S8). For the former case, two FOX-DBD structures in complex with DIV2 DNA (‘DIV’ for diverging half-sites) were reported (17,19). The FOX-DBD molecules exhibited diverged homodimerization harboring PPI between wing 1 regions in each protein (Supplementary Figure S8A). However, our FOXA1-DBD structure was strikingly different from these previously shown structures. The converged dimerization, which refers to the opposite direction of FOXA1-DBD molecules on DNA, was observed, involving unexpected interaction between N-terminal parts of each protein, which was mediated by magnesium ions (Figure 7D). It is of interest that this converged dimerization coincides with the predicted FOXA1 homodimer model in the prostate cancer cell, LNCaP (69), suggesting that the converged binding might be related to biological roles of FOXA1 in cancer cells.

The latter case is DNA-mediated homodimerization, which lacks the role of PPI-mediated allostery (Supplementary Figure S8B). It was shown that two FOXK1a molecules bound to *IL-2* promoter sequences harboring a canonical FOX-binding motif with a direct repeat of forward–forward configuration (38). Our FOXL2-DBD structure in complex with p53-DNA also exhibited a similar binding pattern, except that the binding direction was opposite (reverse–reverse) (Supplementary Figure S8B).

To verify that the FOXL2-DBD molecules lack PPI, we aligned three representative dimerization models (7VOV as a DNA-mediated homodimer, 6LBI as a diverged homodimer with PPI and 7VOX as a converged homodimer with PPI) and compared the position of the second binding site (Figure 6D). When we aligned the three structures by superimposing DBD1 molecules, DBD2 molecules from the diverged model (burgundy) and the converged model (light teal) were adjacent to DBD1 to form a dimer interface. However, FOXL2-DBD2 was positioned opposite to DBD1, rendering them too far apart to make a dimer interface (Figure 6D). Therefore, this structural comparison supports that the DNA groove allostery via minor groove contraction mediates the cooperative binding of FOXL2 to p53-DNA without involving PPI.

Although the FOX family proteins share highly conserved sequences and mainly recognize the DNA via helix 3, they diversify their function using variable regions such as wing 1 or wing 2. This feature was clearly shown in the crystal structures of FOXL2 or FOXA1 in complex with p53-DNA, exhibiting a distinct dimer configuration in recognition of the same p53-DNA (see Supplementary Figure S8). It should be noted that Ser177 in FOXA1 is less conserved above key interaction residues in the Mg^2+^-mediated dimer interface, and FOXL2 or FOXO3 has a distinct residue in this location (Figure 7F). In this regard, this peculiar interaction seems to be a key characteristic which might cause the distinct binding mode of FOXA1 to DNA. These different binding patterns of FOXL2 and FOXA1 to p53-DNA would be another basis to understand how FOX proteins bind to the same DNA in a distinct manner to expand their functional diversity.

Intriguingly, we unveiled a new FOX-binding site (FBE2) on the *TP53* promoter, which emerges via DNA allostery and whose sequence is distinct from that of the typical FOX-binding motif. It is worth noting that the FBE2 site could not be identified by conventional methods such as ChIP-qPCR or *in silico* analyses. As the FBE2 site does not have a measurable binding affinity for FOX proteins prior to the binding of FOX proteins to the neighboring FBE1 site, the FBE2 site would be poorly predicted by similar position weight matrix (PWM) clusters using *in vitro* assays (70,71). In this regard, our structural studies of FOX proteins in complex with the *TP53* promoter provided the first experimental evidence indicating that DNA allostery is a potentially important regulatory mechanism for the non-canonical HCT. The positive cooperativity in HCTs via DNA allostery might be a conserved mechanism for FOX transcription factors, which may be applied to other HCTs in the human genome.

Although the biological role of the non-canonical hHCT is yet to be explored, growing evidence shows that their existence can be implicated in diverse biological functions via different transcriptional control of the same transcription factors (70,72). Even subtle changes in the rate or degree of transcription of transcription factors could have huge biological consequences, as each transcription factor regulates numerous target genes (66). Thus, we hypothesized that the HCT of the *TP53* promoter would contribute to the precise control of p53 expression levels (73,74). As positive cooperative binding leads the affinity curve of a transcription factor to be sigmoidal rather than hyperbolic, it generally helps to respond decisively to a particular range of transcription factor concentrations, enabling fine-tuning control in a narrow concentration range of transcription factors (75–77). Moreover, sigmoidal control can be extended to the construction of biological on/off switches (78). Consistently, cooperativity of binding sites has been suggested as a possible explanation for sigmoidal transcriptional activity (79,80). Also, recent studies emphasized ‘cooperativity’ and affinity of interactions between transcription factors and promoters as important factors for phase separation, which can play a crucial role in transcriptional control (81,82). In these aspects, the homotypic cluster is required to precisely control the p53 expression levels depending on the cellular concentrations of FOX proteins in various tissues. Taken together, our findings indicate that the family of FOX proteins might play a decisive role as an up-regulator of tumor suppressor protein p53 via HCTs for precise control of the *TP53* promoter.

## DATA AVAILABILITY

The crystallographic coordinates of FOXL2-DBD:DBE2 DNA, FOXL2-DBD:p53-DNA and FOXA1-DBD:p53-DNA complex have been deposited in the RCSB Protein Data Bank (www.wwpdb.org) with accession numbers 7VOU, 7VOV and 7VOX, respectively.

## Supplementary Material

gkac673_Supplemental_File
