# Supplementary material for: FOXL2 and FOXA1 cooperatively assemble on the TP53 promoter in alternative dimer configurations
Source: Nucleic Acids Res. 2022 Aug 3;50(15):8929–46. doi: 10.1093/nar/gkac673 (PMC9410875; doi:10.1093/nar/gkac673)
Supplement: gkac673_Supplemental_File [file gkac673_supplemental_file.pdf]

Supplemental Information

FOXL2 and FOXA1 cooperatively assemble on the *TP53* promoter in alternative dimer configurations

Yuri Choi[1,†], Yongyang Luo[2,†], Seunghwa Lee[3], Hanyong Jin[4], Hye-Jin Yoon[1], Yoonsoo Hahn[3], Jeehyeon Bae[2,*], and Hyung Ho Lee[1,*]

[1]Department of Chemistry, College of Natural Sciences, Seoul National University, Seoul 08826, Korea
[2]School of Pharmacy, Chung-Ang University, Seoul 06974, Korea
[3]Department of Life Science, Chung-Ang University, Seoul 06974, Korea
[4]Key Laboratory of Natural Medicines of the Changbai Mountain, Ministry of Education, College of Pharmacy, Yanbian University, Yanji 133002, Jilin Province, China

[†]These authors contributed equally to this work.

*To whom correspondence should be addressed. Tel: +82 2 880 4129; Email: hyungholee@snu.ac.kr
Correspondence may also be addressed to Jeehyeon Bae. Tel: +82 2 820 5604; Email: jeehyeon@cau.ac.kr

This PDF file includes:

Supplementary Figures 1 to 8

Supplementary Table 1 to 3

Supplementary Figures

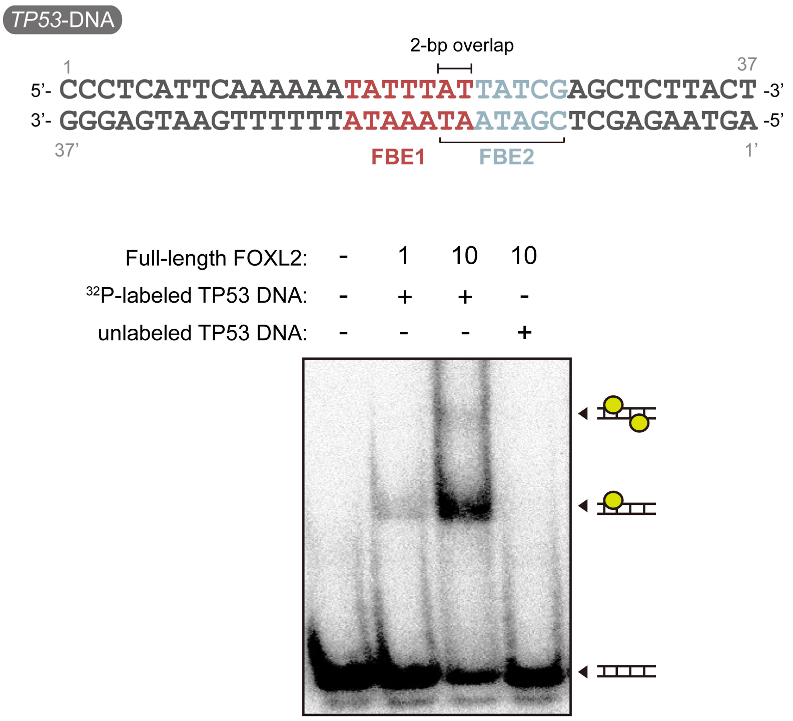

Supplementary Figure 1. Binding of full-length FOXL2 protein with TP53-DNA. The binding was detected by EMSA using recombinant FOXL2 protein (1 μg or 10 μg) and ^{32}P-radiolabeled double strand TP53-DNA. The DNA sequences of *TP53* promoter encompassing the FOXL2 binding elements (FBE1 and 2) are shown on the top. Unlabeled cold probe of TP53-DNA was used as a control. The dimeric protein-DNA complex, monomeric protein-DNA complex, and free DNA are illustrated.

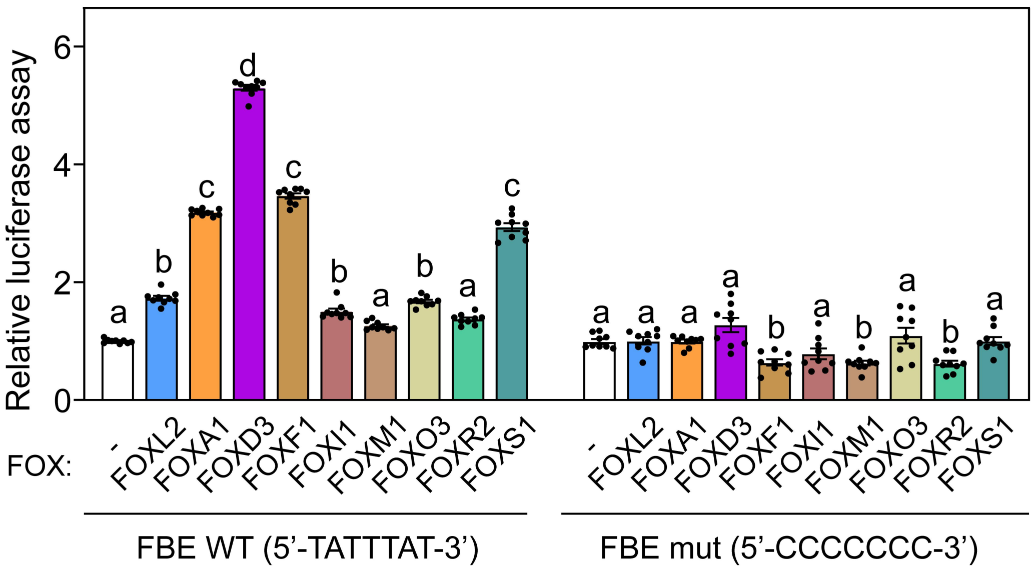

Supplementary Figure 2. Luciferase activity of various FOX proteins on *TP53* promoter. The regulation of *TP53* transcription by FOX proteins was detected by luciferase assay using WT or mutant pGL4.10-TP53 constructs. Data are presented as the mean ± SEM of three independent experiments performed in triplicate. Different letters denote statistically significant differences (p < 0.001).

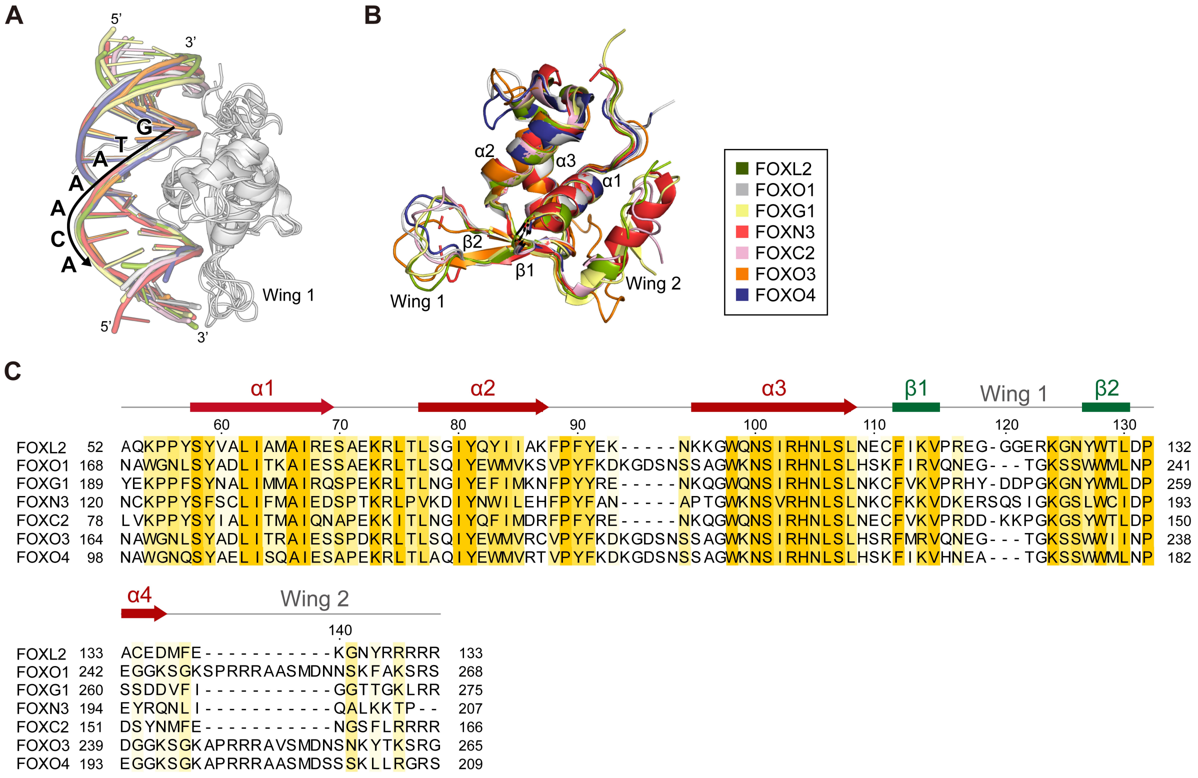

Supplementary Figure 3. Overall comparison of FOX protein structures in complex with DBE2 DNA. **(A-B)** Superimposition between FOXL2 (green, PDB ID: 7VOU), FOXO1 (light gray, PDB ID: 3CO7), FOXG1 (light yellow, PDB ID: 7CBY), FOXN3 (red, PDB ID: 6NCE), FOXC2 (light pink, PDB ID: 6AKO), FOXO3 (orange, PDB ID: 2UZK), and FOXO4 (blue, PDB ID: 3L2C). Typical FOX-binding element (5'-GTAAACA-3') is shown in DNA (left). **(C)** Sequence alignment of FOXL2 from *H. sapiens* (UniProtKB/Swiss-Prot accession number P58012) against FOXO1 from *H. sapiens* (UniProtKB/Swiss-Prot accession number Q12778), FOXG1 from *H. sapiens* (UniProtKB/Swiss-Prot accession number P55316), FOXN3 from *H. sapiens* (UniProtKB/Swiss-Prot accession number O00409), FOXC2 from *H. sapiens* (UniProtKB/Swiss-Prot accession number Q99958), FOXO3 from *H. sapiens* (UniProtKB/Swiss-Prot accession number O43524), and FOXO4 from *H. sapiens* (UniProtKB/Swiss-Prot accession number P98177). Secondary structural elements of FOXL2 were assigned using PyMOL. Red arrows indicate the α-helixes and green

boxes indicate the β-sheets. Yellow color boxes in the sequences indicate the conservation with gradient transparency.

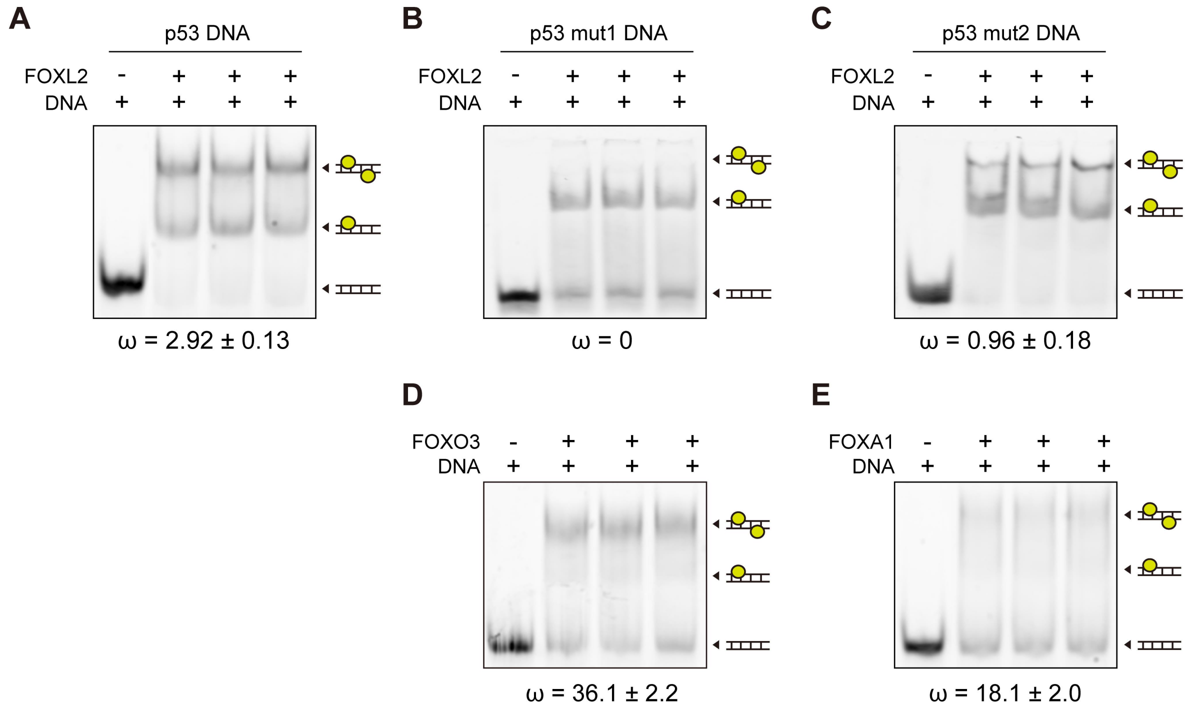

Supplementary Figure 4. Cooperativity factor calculation. **(A-C)** Quantitative EMSA results for FOXL2 incubated with (A) p53 DNA, (B) p53 mut1 DNA, and (C) p53 mut2 DNA using Cy5-labeled p53 DNA. **(D-E)** Quantitative EMSA results for (D) FOXO3 and (E) FOXA1 using Cy5-labeled p53 DNA. Equal amount of DNA and each protein were used in quantitative EMSA. Cooperativity factors (ω) were calculated as previously described.

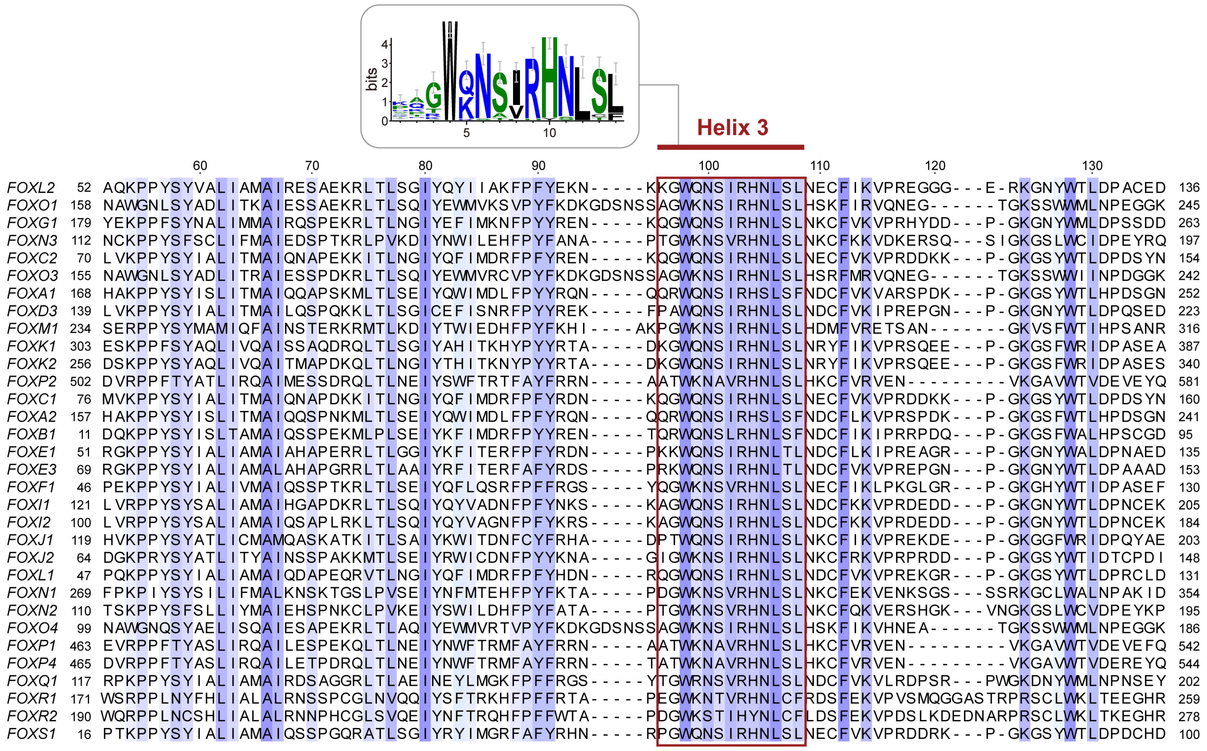

Supplementary Figure 5. Multiple-sequence alignment of a forkhead domain in 32 fox family proteins. Forkhead domain sequences of FOX proteins from *H. sapiens* were compared with those of the FOXL2 protein from *H. sapiens*. Conserved amino acid residues are colored in purple with gradient conservation. Helix 3 sequences in the forkhead domain are highlighted with a burgurdy-colored box, and are also represented in Weblogo 3 with conservation of residues in helix 3.

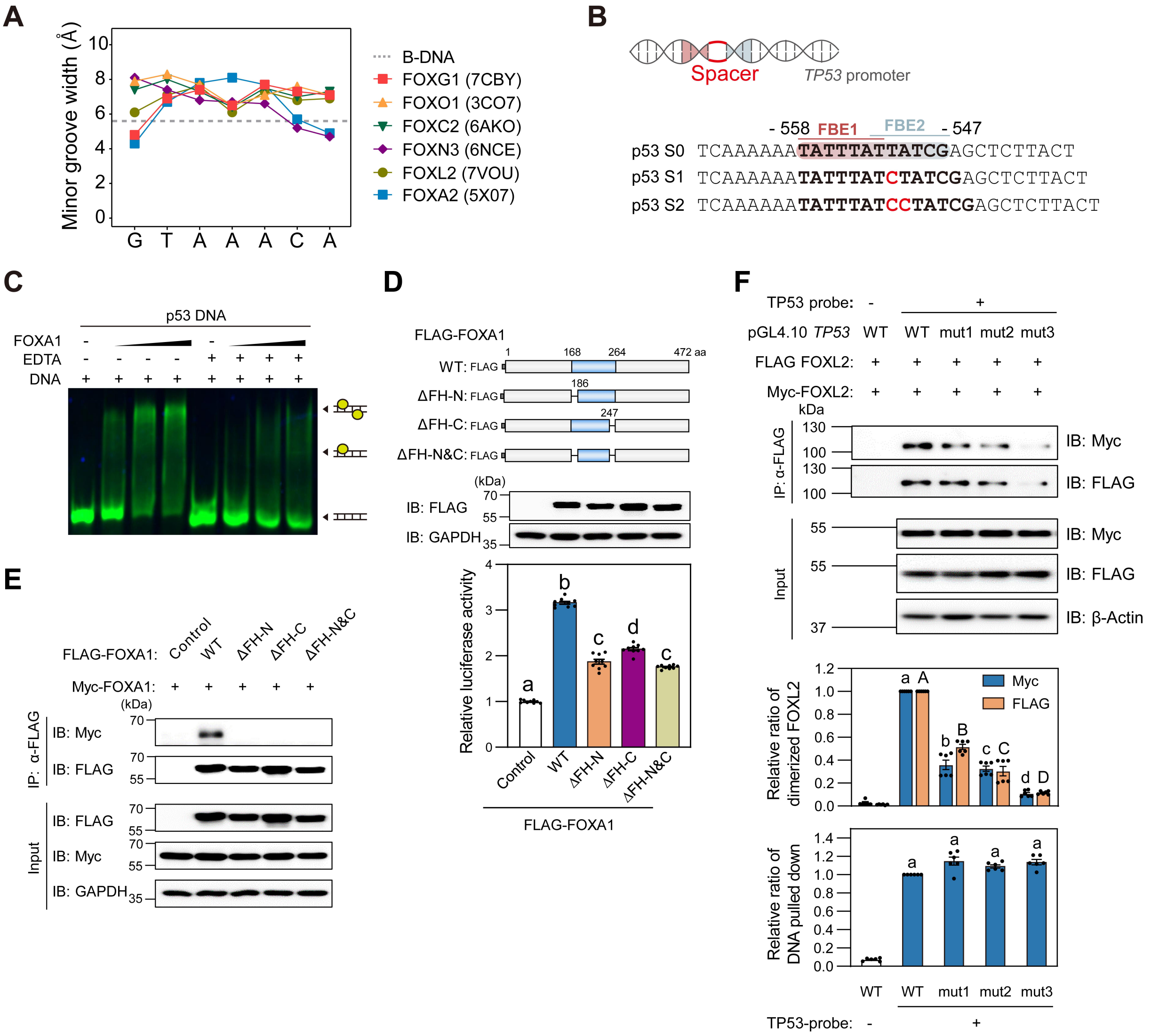

Supplementary Figure 6. Groove parameters of DNA, transcriptional activation of FOXA1 mutants and detection of FOXL2 dimerization. **(A)** Minor groove parameters of typical FOX-binding motifs (5'-GTAAACA-3') in the structure of FOXL2-DBD:DBE2 DNA (PDB ID: 7VOU), FOXG1-DNA (PDB ID: 7CBY), FOXO1-DNA (PDB ID: 3CO7), FOXC2-DNA (PDB ID: 6AKO), FOXN3-DNA (PDB ID: 6NCE), and FOXA2-DNA (PDB ID: 5X07). **(B)** Sequences of p53 WT DNA (S0) and its variants containing 1-bp spacer (S1) and 2-bp spacer (S2) are shown. Inserted spacers are highlighted in red. **(C)** EMSA results of FOXA1-DBD using p53 DNA in Mg^{2+}-depleted condition. 2.5 μM of Cy5-labeled p53 DNA probes were incubated with gradient ratio of FOXA1-DBD, and

excess EDTA was added to reaction buffer for the Mg^{2+}-depleted condition. The dimeric protein-DNA complex, monomeric protein-DNA complex, and free DNA are illustrated. **(D)** Schematic representations of the plasmids encoding full-length (WT) and Forkhead domain (FH) truncated mutants of FOXA1, which were generated to determine the transcriptional activation of *TP53*. Luciferase reporter assays were performed in FOXA1 mutant transfected cells and the equal expression of FOXA1 mutants were determined by western blot. GAPDH was used as a loading control. Data are presented as the mean ± SEM of three independent experiments performed in triplicate. Different letters denote statistically significantly differences (p < 0.05). **(E)** Dimeric FOXA1 detection on *TP53* promoter. Dimeric FOXA1 was detected in 293T cells transfected with Myc-FOXL2 and FLAG-FOXL2 mutants. Cell lysates were immunoprecipatated with α-FLAG antibody followed by immunoblotting with the indicated antibodies. **(F)** Dimeric FOXL2 detection on *TP53* promoter. Myc-FOXL2, FLAG-FOXL2 and TP53 reporter constructs ectopically expressed in cells were cross-linked and *TP53* promoter DNA in cell lysates was pulled down using TP53 DNA probes followed by immunoprecipation with α-FLAG. The representative blots and quantified dimerization ratio are shown. The ratios of dimeric FOXL2 were quantified using the immunoprecipitated FLAG- and Myc-tagged FOXL2. Data are presented as the mean ± SEM of two independent experiments. The pulled down of an equal DNA amount in every group was confirmed by qRT-PCR. Data are presented as the mean ± SEM of two independent experiments performed in triplicate. Different letters denote statistically significantly differences (p < 0.05).

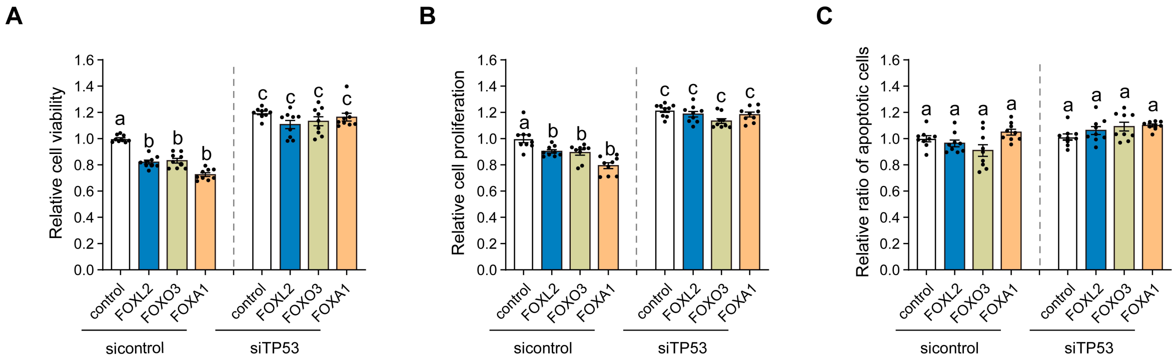

Supplementary Figure 7. The effects of TP53 regulation by FOX proteins in SiHa cells. The effect of FOX proteins on (**A**) cell viability, (**B**) proliferation, and (**C**) apoptosis was examined in control or siTP53-silenced SiHa cells. Data are presented as the mean ± SEM of three independent experiments performed in triplicate. Different letters denote statistically significantly differences (p < 0.05).

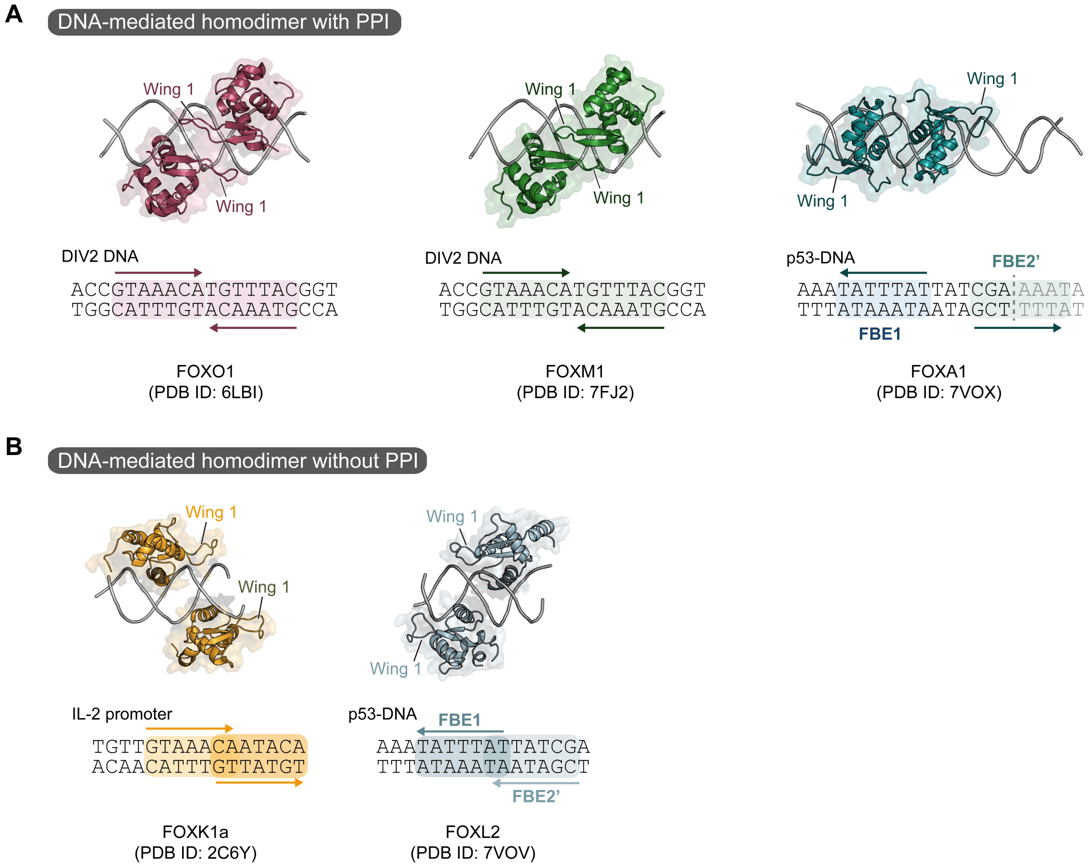

Supplementary Figure 8. Structural comparison between homodimer structures of FOX family proteins. **(A-B)** Homodimer structures of FOX family proteins classified with **(A)** DNA-mediated homodimer with PPI and **(B)** DNA-mediated homodimer without PPI. DNA reading orientations are indicated by arrows. DNA recognition sites are highlighted in distinct colors.

Supplementary Table 1. Primers used in this study.

	DNA	Sequence (5′-3′)
Human *TP53* promoters	-1800F	5´-AAGGTACCCAGCTCCCTGCCCCGTTGTT-3´
	-1800R	5´-TTGCTAGCAACCCCTGCGAGGCTCCTGG-3´
	-551F	5´-AAGGTACCTATCGAGCTCTTACTTGCTA-3´
	-551R	5´-TTGCTAGCAACCCCTGCGAGGCTCCTGG-3´
pGL4.10-TP53 mutant	TP53-mut1-F	5’-CCTGTCTCCCTCATTCAAAAAACCCCCCCTATCG-3’
	TP53-mut1-R	5’-GTGCTGGGTAGCAAGTAAGAGCTCGATAGGGGGG GTTTTTTG-3’
	TP53-mut2-F	5’-CTCCCTCATTCAAAAAATATTTATCCCTCAGCTC-3’
	TP53-mut2-R	5’-GTGCTGGGTAGCAAGTAAGAGCTGAGGGATAAATA TTTTTTG-3’
	TP53-mut3-F	5’-CCTGTCTCCCTCATTCAAAAAACCCCCCCCCCTCA GCTC-3’
	TP53-mut3-R	5’-GTGCTGGGTAGCAAGTAAGAGCTGAGGGGGGGG GGTTTTTTG-3’
pCMV-Myc-FOXL2 mutants	S58A-F	5’- CCGTACGCATACGTGGCGCT-3’
	S58A-R	5’-CACGTATGCGTACGGGGGCT-3’
	Y59A-F	5’-TACTCGGCAGTGGCGCTCAT-3’
	Y59A-R	5’- ATGAGCGCCACTGCCGAGTA-3’
	Y81A-F	5’-GGCATCGCACAGTACATCAT-3’
	Y81A-R	5’-GTACTGTGCGATGCCGGACA-3’
	N100A-F	5’- GAAGGGCTGGCAAGCAAGCATC-3’
	N100A-R	5’- GATGCTTGCTTGCCAGCCCT-3’
	S101A-F	5’- CAAAATGCAATCCGCCACAA-3’
	S101A-R	5’- GCGGATTGCATTTTGCCAGC-3’
	R103A-F	5’- AAATAGCGCACGCCACAACCT-3’
	R103A-R	5’- GTGGCGTGCGCTATTTTGCCAG-3’
	H104A-F	5’- GGCAAAATAGCATCCGCGCAAACCT-3’
	H104A-R	5’- CGTTGAGGCTGAGGTTTGCGCGGAT-3’
	N105A-F	5’- CATCCGCGCAAACCTCAGCC-3’
	N105A-R	5’- GAGGTTCGTGCGGATGCTAT-3’
	S107A-F	5’- CCACAACGCAAGCCTCAACG-3’
	S107A-R	5’- TGAGGCTCGTGTTGTGGCGG-3’
	L108A-F	5’- CAACCTCGCTCTCAACGAGT-3'
	L108A-R	5’- CGTTGAGTGCGAGGTTGTGG-3'

Supplementary Table 1. *contd.*

	DNA	Sequence (5′-3′)
pCMV-Myc-FOXL2 mutants	K124A-F	5’-GAGCGCGCAGGCAACTACTG-3’
	K124A-R	5’- GTTGCCTGCGCGCTCGCCGC-3’
	Helix 1-F	5’-CCGTACGCAGCAGTGGCGCTCAT-3’
	Helix 1-R	5’- CGCCACTGCTGCGTACGGGG-3’
	Helix 3-F	5’-GAATAAGAAGGGCTGGCAAGCAGCAATCGCAG CAGCACTCGCAGCAAACGAGT-3’
	Helix 3-R	5’- CCTTGATGAAGCACTCGTTTGCTGCGAGTGCTGC TGCGATTGCTGCTTGCCAG-3’
p3XFLAG-CMV-10-FOXL2	FOXL2-F	5’- GCGAATTCAATGATGGCCAGCTACCCCGA-3’
	FOXL2-R	5’-CTGGTACCTCAGAGATCGAGGCGCGAAT-3’
-FOXA1	FOXA1-F	5’- GCGAATTCAATGTTAGGAACTGTGAAGATGG-3’
	FOXA1-R	5’- CTGGTACCCTAGGAAGTGTTTAGGACGG-3’
-FOXO3	FOXO3-F	5’-TCGGTACCAATGGCAGAGGCACCGGCTTC-3’
	FOXO3-R	5’-CGGGATCCTCAGCCTGGCACCCAGCTCTGA-3’
p3XFLAG-CMV-10-FOXA1 mutants	Y173A, S174A, S177A-F	5’-CCGCCCGCAGCATACATCGCACTCATCAC CATGGCCATCCA-3’
	Y173A, S174A, S177A-R	5’-GATGAGTGCGATGTATGCTGCGGGCGGCTT GGCGTGCGGGT-3’
	ΔFH-N-F	5’-GCTACCCGGCGCCCAGCAAGATGCTCAC-3’
	ΔFH-N-R	5’-CTGGGCGCCGGGTAGCTGCGCTTGAACG-3’
	ΔFH-C-F	5’-GCTGCACCGCTTCAAGTGCGAGAAGCA-3’
	ΔFH-C-R	5’-TTGAAGCGGTGCAGCGTCCAGTAGGAGC-3’
Real-time PCR	TP53-F	5´-GAGGTTGGCTCTGACTGTACC-3´
	TP53-R	5´-TCCGTCCCAGTAGATTACCAC-3´
	GAPDH-F	5’-AGGGGCCATCCACAGTCTT-3’
	GAPDH-R	5’- AGCCAAAAGGGTCATCATCTCT-3’
pLenti-puro FLAG-tagged FOXL2	FLAG-FOXL2-F	5´-TTGGATCCGCCACCATGGCATCAATGCAGAA-3´
	FLAG-FOXL2-R	5´-AATCTAGAAGTCAGAGATCGAGGCGCGAA-3´
ChIP analysis	FBE-F	5´-TCTCATTCTCCAGGCTTCAGA-3´
	FBE-R	5´-TAGAATTTTTCTACTATCTTA-3´
pGST2-FOXL2	FOXL2-52F	5’-GCTATATGGATCCGCGCAGAAGCCCCCGTAC-3’
	FOXL2-148R	5’-GCTAATTCTCGAGTCAGCGGCGGCGGCGCCGG-3’
-FOXA1	FOXA1-168F	5’-GCTATATGGATCCCACGCCAAGCCGCCCTAC-3’
	FOXA1-264R	5’-GCTAATTCTCGAGTCACTTCTGGCGGCGCAAGTAG-3’
-FOXO3	FOXO3-155F	5’-GCTATATGGATCCAACGCCTGGGGAAACC-3’
	FOXO3-251R	5’-GCTAATTCTCGAGTCAAGCCCGCCGCCGGGGG-3’

Supplementary Table 1. *contd.*

	DNA	Sequence (5′-3′)
Crystallization	DBE2-F	5’-CAAAATGTAAACAAGT-3’
	DBE2-R	5’- ACTTGTTTACATTTTG-3’
	p53_16bp-F	5’- AAATATTTATTATCGA-3’
	p53_16bp-R	5’-TCGATAATAAATATTT-3’
EMSA	DBE2- F	5’-Cy3-CAAAATGTAAACAAGT-3’
	DBE2-R	5’- ACTTGTTTACATTTTG-3’
	p53_26bp-F	5’-Cy3-TCAAAAAAATATTTATTATCGAGCTCT-3’
	p53_26bp-R	5’- AGAGCTCGATAATAAATATTTTTTTGA-3’
	p53_mut1-F	5’-Cy3-TCAAAAAAACCCCCCCTATCGAGCTCT-3’
	p53_mut1-R	5’- AGAGCTCGATAGGGGGGGTTTTTTGA-3’
	p53_mut2-F	5’-Cy3-TCAAAAAAATATTTATCCCTCAGCTCT-3’
	p53_mut2-R	5’- AGAGCTGAGGGATAAATATTTTTTTGA-3’
	p53_S1-F	5’-Cy3-TCAAAAAAATATTTATCTATCGAGCTCT-3’
	p53_S1-R	5’-AGAGCTCGATAGATAAATATTTTTTTGA-3’
	p53_S2-F	5’-Cy3-TCAAAAAAATATTTATCTATCGAGCTCT -3’
	p53_S2-R	5’-AGAGCTCGATAGATAAATATTTTTTTGA-3’

Supplementary Table 2. Double-strand DNAs used in this study.

	DNA	Sequence (5′-3′)
Crystallization	DBE2	CAAAATGTAAACAAGT
	p53	AAATATTTATTATCGA
ITC	p53	AAATATTTATTATCGA
	p53 mut1	AAACCCCCCCTATCGA
	p53 mut2	AAATATTTATCCCTCA
EMSA	DBE2	CAAAATGTAAACAAGT
	p53	TCAAAAAATATTTATTATCGAGCTCT
	p53 mut1	TCAAAAAACCCCCCCTATCGAGCTCT
	p53 mut2	TCAAAAAATATTTATCCCTCAGCTCT
	p53 S1	TCAAAAAATATTTATCTATCGAGCTCT
	p53 S2	TCAAAAAATATTTATCCTATCGAGCTCT

Supplementary Table 3. Crystallographic data collection and refinement statistics.

Data set	FOXL2-DBE2 DNA	FOXL2-p53 DNA	FOXA1-p53 DNA
PDB codes	7VOU	7VOV	7VOX
A. Data collection			
X-ray source	PLS-5C	PLS-5C	PLS-5C
X-ray wavelength (Å)	1.0000	0.9795	0.9794
Space group	$P4_12_12$	$P3_121$	$C222$
Unit cell length (*a*, *b*, *c*, Å)	48.0, 48.0, 164.4	98.6, 98.6, 64.7	138.5, 144.3, 75.0
Unit cell angle (α, β, γ, °)	90, 90, 90	90, 90, 120	90, 90, 90
Resolution range (Å)	50.0–3.10 (3.15–3.10)[a]	50.0–3.14 (3.32–3.14)[a]	50.0–2.10 (2.14–2.10)[a]
Total / unique reflections	329,482 / 3,962	131,134 / 6,586	1,393,579 / 44,252
Completeness (%)	100.0 (100.0)[a]	99.6 (100.0)[a]	100.0 (100.0)[a]
Average I/σ (I)	31.5 (4.9)[a]	37.0 (6.41)[a]	34.9 (3.3)[a]
R_{merge}[b] (%)	10.0 (91.5)[a]	6.8 (42.8)[a]	11.3 (65.3)[a]
B. Model refinement statistics			
Resolution range (Å)	46.07–3.10	19.77-3.15	50.0-2.10
R_{work} / R_{free}[c] (%)	22.8 / 28.5	24.9 / 27.5	18.6 / 21.2
Monomers per asymmetric unit	1	1	2
Number of non-hydrogen atoms / average *B*-factor ($Å^2$)			
Protein	706 / 92.0	1,499 / 93.3	2,807 / 33.8
DNA	651 / 84.7	635 / 84.9	1,312 / 34.9
Water	0 / 0	0 / 0	446 / 37.9
Mg^{2+}			2 / 9.4
R.m.s. deviations from ideal geometry			
Bond lengths (Å) / bond angles (°)	0.012 / 1.33	0.003 / 0.63	0.005 / 0.81
Protein-geometry analysis			
Ramachandran favored (%)	92.9	93.8	97.9
Ramachandran allowed (%)	7.1	6.2	2.1
Ramachandran outliers (%)	0	0	0

Footnotes for Supplementary Table 3

[a]Values in parentheses refer to the highest resolution shell.

[b] $R_{merge} = \Sigma_{hkl}\Sigma_i \mid I_i(hkl) - \langle I(hkl) \rangle \mid / \Sigma_{hkl}\Sigma_i I_i(hkl)_i$, where $I(hkl)$ is the intensity of reflection *hkl*, Σ_{hkl} is the sum over all reflections, and Σ_i is the sum over i measurements of reflection *hkl*.

[c]$R = \Sigma_{hkl} \mid |F_{obs}| - |F_{calc}| \mid / \Sigma_{hkl} |F_{obs}|$, where R_{free} was calculated for a randomly chosen 5% of reflections, which were not used for structure refinement and R_{work} was calculated for the remaining.
